# Uncovering the Cryptic Gene Cluster *ahb* for 3-amino-4-hydroxybenzoate Derived Ahbamycins, by Searching SARP Regulator Encoding Genes in the *Streptomyces argillaceus* Genome

**DOI:** 10.3390/ijms24098197

**Published:** 2023-05-03

**Authors:** Suhui Ye, Brian Molloy, Ignacio Pérez-Victoria, Ignacio Montero, Alfredo F. Braña, Carlos Olano, Sonia Arca, Jesús Martín, Fernando Reyes, José A. Salas, Carmen Méndez

**Affiliations:** 1 Departamento de Biología Funcional e Instituto Universitario de Oncología del Principado de Asturias (I.U.O.P.A), Universidad de Oviedo, 33006 Oviedo, Spain; 2 Instituto de Investigación Sanitaria de Asturias (ISPA), 33011 Oviedo, Spain; 3 Fundación MEDINA, Centro de Excelencia en Investigación de Medicamentos Innovadores en Andalucía, Armilla, 18016 Granada, Spain

**Keywords:** 3,4-AHBA, *Streptomyces*, SARP, ahbamycins, genome mining, argimycin P, phenoxazinone, aminobenzoate, specialized metabolites, natural products

## Abstract

Genome mining using standard bioinformatics tools has allowed for the uncovering of hidden biosynthesis gene clusters for specialized metabolites in *Streptomyces* genomes. In this work, we have used an alternative approach consisting in seeking “*Streptomyces* Antibiotic Regulatory Proteins” (SARP) encoding genes and analyzing their surrounding DNA region to unearth cryptic gene clusters that cannot be identified using standard bioinformatics tools. This strategy has allowed the unveiling of the new *ahb* cluster in *Streptomyces argillaceus*, which had not been retrieved before using antiSMASH. The *ahb* cluster is highly preserved in other *Streptomyces* strains, which suggests a role for their encoding compounds in specific environmental conditions. By combining overexpression of three regulatory genes and generation of different mutants, we were able to activate the *ahb* cluster, and to identify and chemically characterize the encoded compounds that we have named ahbamycins (AHBs). These constitute a new family of metabolites derived from 3-amino-4-hydroxybenzoate (3,4-AHBA) known for having antibiotic and antitumor activity. Additionally, by overexpressing three genes of the cluster (*ahbH*, *ahbI,* and *ahbL2*) for the synthesis and activation of 3,4-AHBA, a new hybrid compound, AHB18, was identified which had been produced from a metabolic crosstalk between the AHB and the argimycin P pathways. The identification of this new BGC opens the possibility to generate new compounds by combinatorial biosynthesis.

## 1. Introduction

Natural products, also known as specialized metabolites (SM) [1], are the richest source of bioactive compounds used in medicine, livestock, and agriculture [2]. Among microorganisms, bacteria belonging to the *Streptomyces* genus stand out for producing the highest percentage of SM, and about 68% of the most important bioactive SM [3]. Since the first *Streptomyces* genomes were sequenced [4,5], it became clear that these bacteria have the capacity to encode far more biosynthesis gene clusters (BGCs) for SM than was initially expected. Over the last twenty years, several bioinformatics tools have been developed to search for BGCs including the Antibiotic and Secondary Metabolites Analysis Shell (antiSMASH) [6,7,8]. This tool has greatly facilitated identifying a huge number of BGCs in *Streptomyces*, which highlighted the potential of their genomes to encode a vast number of bioactive compounds to be discovered. Most of the BGCs identified so far encode polyketide and/or peptide derived compounds [9]. However, novel classes of BGCs encoding other SMs remain difficult to be identified although some approaches have been developed [10,11]. 

BGC-associated genes typically include those encoding biosynthetic enzymes, self-resistance systems, and transport-related proteins. In addition, most BGCs contain genes for “Cluster-Situated Regulators” (CSR) [12] that together with a complex network of global regulatory genes, regulate the expression of other genes within BGCs [13,14,15]. The so-called “*Streptomyces* Antibiotic Regulatory Proteins” (SARPs) [16] are the CSR most frequently found in *Streptomyces* BGCs, which are usually functioning as pathway-specific activators [13]. An approach to uncover novel classes of BGCs could be the use of SARP encoding genes as a genome mining hook, since this type of genes are often located at BGCs [13].

*Streptomyces argillaceus* ATCC 12956 is the producer of the known antitumor compound mithramycin. Its genome has been sequenced [17] and mined for BGCs using antiSMASH analysis [6,8]. This has allowed identifying 31 BGCs, some of which have been already characterized. These include the previously characterized BGC for the antitumor mithramycin (*mtm*) [18]; four silent BGCs encoding desferrioxamine (*desa*), carotenoids (*crta*), germicidins (*gcs*), and antimycins (*anta*) [19]; and two cryptic BGCs, which encode the biosynthesis of argimycins P (*arp*) [17] and largimycins (*lrg*) [20]. The aim of this work was to test searching SARP encoding genes as a strategy to identify new BGCs that are not detected using antiSMASH. We report the identification of SARP genes in *S. argillaceus* genome to uncover the previously unidentified *ahb* BGC, its activation by overexpressing three CSR genes, and the identification and chemical characterization of their encoded compounds, the ahbamycins (AHBs), which constitute a new group of metabolites derived from 3,4-AHBA known for having antibiotic and antitumor activity. Following this strategy, we have additionally generated a new hybrid compound produced from the metabolic crosstalk between the AHB and the argimycin P (ARP) pathways.

## 2. Results

### 2.1. Identification of the ahb Biosynthesis Gene Cluster

**Figure 1:**
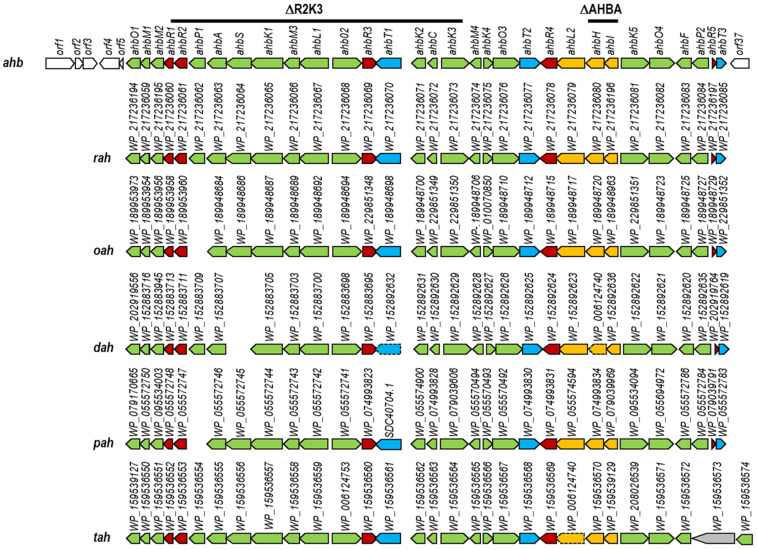
Genetic organization of cluster *ahb* from *Streptomyces* argillaceus, and comparison to homologous clusters in other *Streptomyces* strains. Clusters shown are *ahb* from *S. argillaceus*; *rah* from *Streptomyces* sp. AC555_RSS877 (NZ_JAGMUK010000018.1); *oah* from *S. roseolus* JCM 4411 (NZ_BMTV01000035.1; NZ_BMTV01000014.1); *dah* from *S. adustus* NBRC 109810 (NZ_VJZD01000001.1; NZ_VJZD01000132.1); *pah* from *S. prasinopilosus* CGMCC 4.3504 (NZ_FMZK01000002.1); and *tah* from *Streptomyces* sp. Tü 3180 (NZ_WOXS01000002.1). Genes belonging to the BGCs are colored: yellow, 3,4-AHBA biosynthesis genes; green, other biosynthesis genes; red, regulatory genes; blue, transport-related genes; grey, additional genes; white, genes not belonged to the clusters. Arrows with dashed lines indicate genes not wholly sequenced. Genes are shown to scale. Bars indicate DNA regions that have been deleted in *S. argillaceus* mutants.

The *S. argillaceus* genome has been previously sequenced and mined using the antiSMASH bioinformatics tool version 3.0.2 [6,8], which has allowed identifying 31 BGCs [17]. The newest version of that program (version 6.0) [7] identified the same number of BGCs, although with some differences (see Table S1 in Supplementary Materials): version 3.0.2 predicted two unknown BGCs (cluster three and four), while version 6.0 predicted the existence of only one BGC at those positions (cluster four; NRPS-like). In addition, version 6.0 predicted the existence of a BGC (cluster 21) that had not been identified before. In an attempt to identify additional BGCs in *S. argillaceus* genome undetected with any antiSMASH version, we have mined its genome looking for SARP encoding genes using Curated Blast software [21]. Eleven SARP genes were identified, four located at BGCs (*mtm*, *arp*, and BGC 28), and seven outside any BGC previously identified by antiSMASH. Analysis of the DNA region surrounding two of these SARP-encoding genes closely located at the chromosome allowed us to identify a new BGC (named *ahb*, see below) not uncovered in previous analyses (Figure 1). This new cluster was located between clusters 17 and 18 (Table S1) detected by antiSMASH, at the right arm of the chromosome. To determine if similar gene clusters were present in other *Streptomyces* strains and to propose limits to *ahb* BGC, each *ahb* gene product was compared with others in databases using BlastP analyses [22] to identify similar proteins. Then, those coding genes were located in the corresponding *Streptomyces* genomes, and their positions were identified to determine if they were clustered in the same DNA region. In this way, five BGCs were identified (named as *rah*, *oah*, *dah*, *pah*, and *tah*, in Table S2 and Figure 1) in different *Streptomyces* strains, which showed high similarity to the *ahb* BGC: *rah* from *Streptomyces* sp. AC555_RSS877 (NZ_JAGMUK010000018.1: nucleotides 10109 to 46535); *oah* from *S. roseolus* JCM 4411 (NZ_BMTV01000035.1: nucleotides 66046 to 66695; NZ_BMTV01000014.1: nucleotides 1 to 30426); *dah* from *S. adustus* NBRC 109810 (NZ_VJZD01000001.1: nucleotides 222 to 14383; NZ_VJZD01000132.1: nucleotides 5002 to 25986); *pah* from *S. prasinopilosus* CGMCC 4.3504 (NZ_FMZK01000002.1: nucleotides 53452 to 89451); and *tah* from *Streptomyces* sp. Tü 3180 (NZ_WOXS01000002.1: nucleotides 6715158 to 6753232). All these clusters showed high synteny to cluster *ahb*, displaying the same gene organization, and only showing few differences: the absence of one or two genes in some BGCs (*ahbP1* homologous gene was absent in *oah* and *pah*; *ahbS* in *dah*) or the presence of an additional gene in *tah* BGC (WP_159536573). Based on these comparisons, *ahbO1* and *ahbT3* are proposed as the limits of the *ahb* cluster. A phylogenetic analysis of the above-mentioned *Streptomyces* strains was carried out based on 82 housekeeping genes [23] (see Figure S1 in Supplementary Materials). This analysis indicated that those strains were not closely related among them.

**Figure 2:**
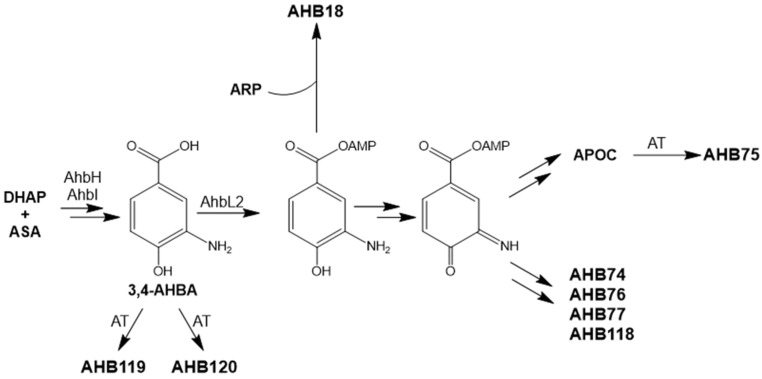
Proposed biosynthesis pathway for ahbamycins. ASA, aspartate semialdehyde; DAHP, dihydroxyacetone-P; AT, acyltransferase.

The *ahb* cluster would span 36.48 kb and contain 31 open reading frames (*orfs*) (Figure 1 and Table S2). It would include genes for the biosynthesis of 3-amino-4-hydroxybenzoic acid (3,4-AHBA) (*ahbH* and *ahbI*); acyl-CoA ligases (*ahbL1* and *ahbL2*); oxygenases (*ahbO1* to *ahbO4*); reductases (*ahbK1* to *ahbK5*); methyltransferases (*ahbM1* to *ahbM4*); carbohydrate kinases (*ahbP1* and *ahbP2*); a cupin domain-containing protein (*ahbC*); a lyase (*ahbS*); proteins related to an activated methyl-cycle (*ahbA* and *ahbF*); regulatory proteins (*ahbR1* to *ahbR5*); and transport proteins (*ahbT1* to *ahbT3*). The presence of *ahbH* and *ahbI* suggested that compounds encoded by these *ahb* BGCs contained a 3,4-AHBA moiety [24] (Figure 2; see Section 3). 

### 2.2. Identification of Compounds Encoded by the ahb Cluster

To identify compounds encoded by *ahb*, several approaches were tried. The first was the generation of two independent deleted mutants in which a DNA region from the *ahb* BGC was replaced by an apramycin resistance gene (Figure 1): (i) *S. argillaceus* ΔAHBA. Using pHZΔasu (Table 1) genes *ahbH* and *ahbI* were deleted and replaced by an apramycin resistance cassette; and (ii) *S. argillaceus* ΔR2K3 in which genes from *ahbR2* to *ahbK3* were deleted by using pHZΔasu1705 (Table 1). The genotypes of these two mutant strains were confirmed by PCR (see Figures S2 and S3 in Supplementary Materials). Comparison of UPLC chromatograms of broth extracts from the wild type strain and from these two mutants did not show any differential peak, suggesting that cluster *ahb* was silent. 

**Figure 3:**
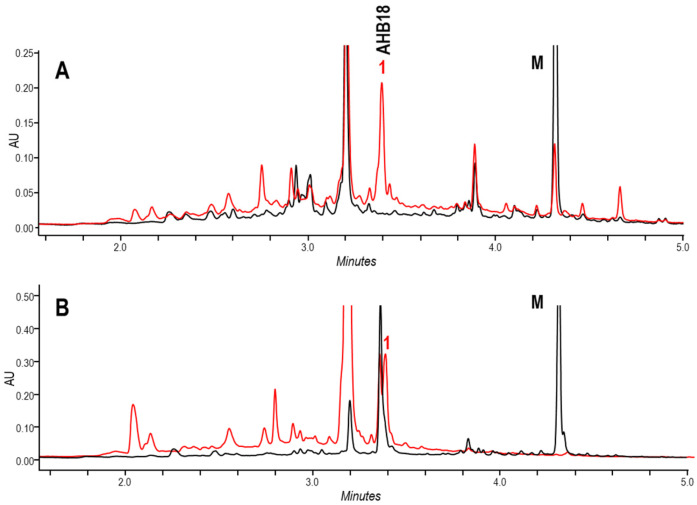
Effect of 3,4-AHBA on ahbamycins production by *S. argillaceus*. UPLC chromatograms (360 nm) of extracts of cultures in R5A medium of (**A**) *S. argillaceus* wild type strain overexpressing genes encoding enzymes for the biosynthesis of 3,4-AHBA (pEM4T-AHBA) (red line), in comparison to *S. argillaceus* wild type strain containing the vector (pEM4T) (black line); and (**B**) *S. argillaceus* wild type (red line) and mutant *S. argillaceus* ΔR2K3 (black line) strains, fed with 3,4-AHBA. M, mithramycins. Peak 1 corresponds to ahbamycin 18 (AHB18).

**Figure 4:**
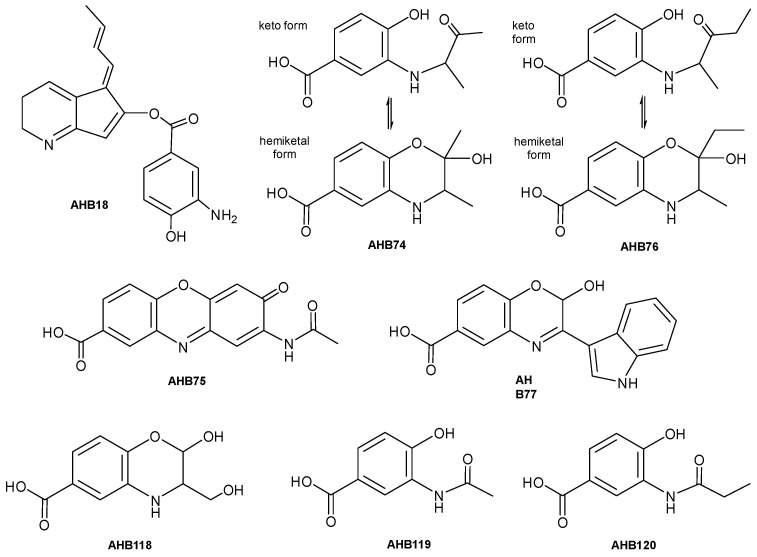
Chemical structures of ahbamycins (AHB).

Secondly, three genes (*ahbI*, *ahbH*, and *ahbL2*) were overexpressed in *S. argillaceus* wild type strain using pEM4T-AHBA (Table 1). These genes would encode enzymes for the biosynthesis of 3,4-AHBA (AhbH and AhbI) and for its activation by adenylation (AhbL2). UPLC analyses of metabolite profiles of cultures from *S. argillaceus* (pEM4T-AHBA) and *S. argillaceus* pEM4T (as control) extracted with *n*-butanol or ethyl acetate, revealed several peaks that were present in the former and absent in the latter (Figure 3A). Some of these compounds were also produced when cultures of *S. argillaceus* wild type strain were fed with 3,4-AHBA, while those that were absent in cultures of *S. argillaceus* ΔR2K3 were fed with the same compound (Figure 3B). This suggested that those differential peaks contained compounds derived from 3,4-AHBA, whose biosynthesis would require the genes deleted in *S. argillaceus* ΔR2K3. The major compound **1** detected in *n*-butanol extracts at 360 nm was purified from cultures of *S. argillaceus* (pEM4T-AHBA) grown in R5A, and structurally characterized (see below). It corresponded to a hybrid new compound constituted by two moieties, a 3,4-AHBA and an ARP compound [17,25,26], and it was given the name ahbamycin 18 (AHBA18) (Figure 4). 

The third approach was overexpression of regulatory genes from the *ahb* cluster. Individual overexpression of *ahbR2* and *ahbR3* that encode AfsR/SARP putative transcriptional activators did not result in production of any differential compound. Then, we jointly co-expressed both SARP-encoding genes, and *ahbR4* that encodes a protein with Ada_Zn_binding and HTH_ARAC domains and is located downstream of the 3,4-AHBA encoding genes. These three regulatory genes were cloned under the control of the erythromycin resistance promoter, and the resultant plasmid pREGT (Table 1) was introduced in *S. argillaceus* WT, *S. argillaceus* ΔAHBA, and *S. argillaceus* ΔR2K3. The resultant recombinant strains (*S. argillaceus* WT-pREGT; *S. argillaceus* ΔAHBA-pREGT; and *S. argillaceus* ΔR2K3-pREGT) were cultivated in R5A and SM10 media, and cultures were extracted with different organic solvents.

**Figure 5:**
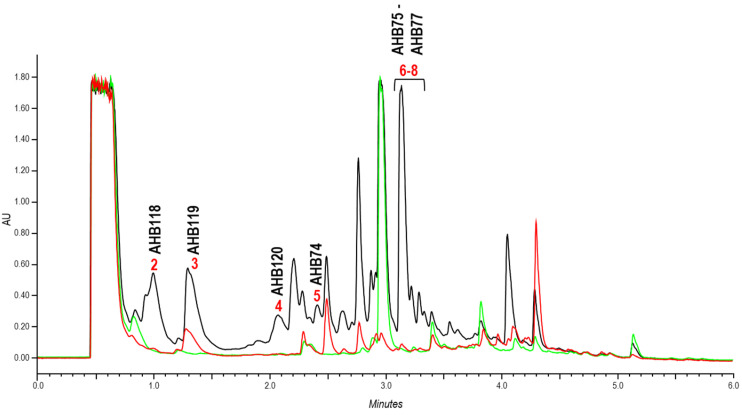
Production of ahbamycins by *S. argillaceus* strains overexpressing regulatory genes. UPLC chromatograms (230 nm) of extracts of *S. argillaceus* WT-pREGT (black line), *S. argillaceus* ΔAHBA-pREGT (green line), and *S. argillaceus* ΔR2K3-pREGT (red line), cultivated in SM10. Peaks with numbers correspond to those ahbamycins (AHB) selected for chemical characterization. Peaks for AHB118, AHB119, AHB120, AHB74, and AHB75 to AHB77 are indicated.

In all culture and extraction conditions, several peaks were detected in extracts from *S. argillaceus* WT-pREGT that were absent in those from *S. argillaceus* ΔR2K3-pREGT and/or *S. argillaceus* ΔAHBA-pREGT (Figure 5). Production of those peaks was recovered in *S. argillaceus* ΔAHBA-pREGT when the deleted genes in this strain were expressed *in trans* using plasmid pSETeAHBAHyg (Table 1; see Figure S4). This confirmed the involvement of *ahb* BGC in the production of those differential peaks. These new compounds were produced better in SM10 cultures, and best extracted with ethyl acetate containing 1% formic acid. Consequently, these conditions were used thereafter. Compounds from peaks **2**–**8** were purified from *S. argillaceus* WT-pREGT and were named AHB74 to AHB77 (peaks **5** to **8**), and AHB118 to AHB120 (peaks **2** to **4**) (Figure 4 and Figure 5).

### 2.3. Structural Elucidation and Bioactivity of Ahbamycins 

The structure of the isolated ahbamycins was established by UV/vis (DAD), MS, and NMR spectroscopic analyses (see Supplementary Materials). 

**Figure 6:**
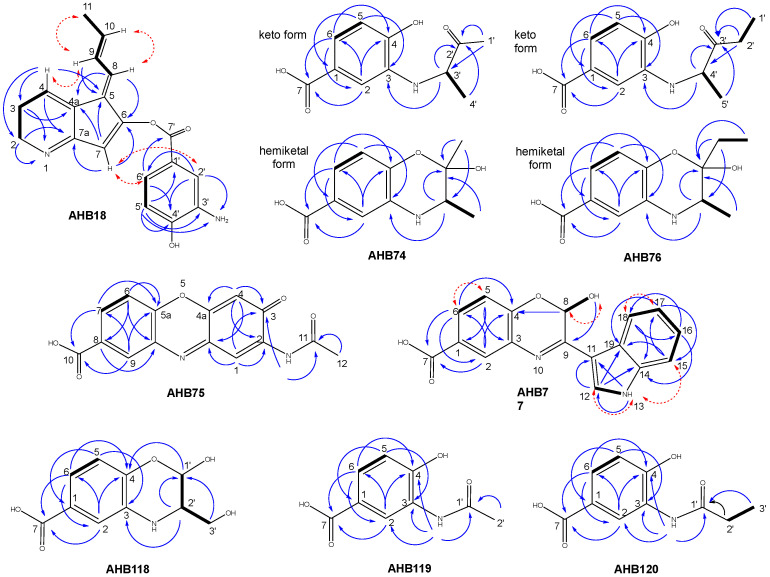
Key COSY (bold bonds), HMBC (blue arrows), and NOESY (dashed red arrows) correlations used to determine the chemical structure of ahbamycins.

AHB18 was assigned the molecular formula C_19_H_18_N_2_O_3_ based on the observed [M + H]^+^ ion at *m*/*z* = 323.1396 (calcd. for C_19_H_19_N_2_O_3_^+^ = 323.1390, Δ = 1.9 ppm), indicating twelve degrees of unsaturation. Its UV/vis (DAD) spectrum suggested the presence of an aromatic or conjugated *π* system. Analysis of the ^1^H and HSQC NMR spectra indicated a total of fifteen non-exchangeable hydrogens. The HSQC spectrum revealed the presence of one aliphatic methyl group, two methylenes (one likely bound to nitrogen), and eight sp^2^ methines. Such structural features are not compatible with any of the compounds included in the Dictionary of Natural Products [27] having the same molecular formula, confirming the novelty of the compound. Additional 2D NMR spectra, including COSY, NOESY, HMBC (the standard ^1^H-^13^C), and ^1^H-^15^N HMBC, were acquired to determine the compound structure. The key correlations observed in the COSY spectrum identified the different spin systems, which were connected via the long-range correlations observed in both the ^1^H-^13^C HMBC and ^1^H-^15^N HMBC spectra (Figure 6), which likewise unambiguously determined the position of the enolic oxygen and amino groups to finally establish the connectivity of the compound. Key NOESY correlations (Figure 6) allowed determining an E stereochemistry for both bonds in the exocyclic chain (Δ^5,8^ and Δ^9,10^) and further supported the proposed connectivity. The compound contains a 3,4-AHBA moiety interestingly conjugated with an ARP subunit, confirming the proposed existence of crosstalk between their two biosynthetic pathways. 

AHB74 was assigned the molecular formula C_11_H_13_NO_4_ based on the observed [M + H]^+^ ion at *m*/*z* = 224.0918 (calcd. for C_11_H_14_NO_4_^+^ = 224.0917, Δ = 0.4 ppm), indicating six degrees of unsaturation. Its UV/vis (DAD) spectrum suggested the presence of an aromatic or conjugated *π* system. 1D ^1^H and 2D NMR spectra (including COSY, HSQC, and HMBC) were acquired to determine the structure of the purified compound. Surprisingly, the ^1^H and HSQC revealed more than a single component in the sample. A possible equilibrium in solution between different interconverting species (in slow exchange on the NMR time scale) would account for such observation. Detailed analysis of the NMR spectra revealed this hypothesis to be correct. To sort out the chemical structure of such equilibrating species, the elucidation started from the aliphatic methine observed at δ_H_ 4.12, δ_C_ 58.1 ppm. Observed key COSY and HMBC correlations (Figure 6) indicated that such methine is directly bound to a methyl group, a carbonyl (from a methyl ketone) and, based on its ^13^C chemical shift, a nitrogen. This nitrogen corresponds to the amino functionality of a 3,4-AHBA moiety according to the key 2D NMR correlations observed in the 2D spectra (Figure 6). The determined connectivity of the first component possesses one chiral center whose stereochemistry remained undetermined. Once we established the structure of this component, it was easier to determine that the other sets of signals correspond to the two possible epimers that originate in the cyclization via nucleophilic attack of the phenol group of the 3,4-AHBA subunit to the ketone carbonyl, rendering two diastereomeric hemiketals whose connectivity was unambiguously corroborated by the observed key COSY and HMBC correlations (Figure 6). The presence of a hemiketal functionality in the molecules is additionally corroborated by the existence of ^13^C signals at 96.4 and 98.5 ppm corresponding to both stereoisomers at C-2′. A stereospecific assignment of the signals of each epimer was not carried out.

AHB75 was assigned the molecular formula C_15_H_10_N_2_O_5_ based on the observed [M + H]^+^ ion at *m*/*z* = 299.0662 (calcd. for C_15_H_11_N_2_O_5_^+^ = 299.0662, Δ = 0 ppm), indicating twelve degrees of unsaturation. Its UV/vis (DAD) spectrum alongside the molecular formula suggested a high degree of conjugation. A search of the molecular formula in the Dictionary of Natural Products [27] retrieved just four possible candidates, all of them compatible with the UV/vis spectrum obtained. To ensure successful dereplication, a set of NMR spectra (^1^H, COSY, HSQC, and HMBC) was acquired. Analysis of the ^1^H and HSQC NMR spectra indicated a total of eight non-exchangeable hydrogens corresponding to five sp2 methines and one aliphatic methyl group from an acetyl substituent according to its chemical shift. These features are compatible with the structure of carboxyexfoliazone [28], also known as umicyn A [29], which interestingly contains a 3,4-AHBA substructural moiety. Analysis of the key correlations observed in the COSY and HMBC spectra (Figure 6) and comparison with the reported NMR data for carboxyexfoliazone [28] unambiguously confirmed its identity.

AHB76 was assigned the molecular formula C_12_H_15_NO_4_ based on the observed [M + H]^+^ ion at *m*/*z* = 238.1075 (calcd. for C_12_H_16_NO_4_^+^ = 238.1074, Δ = 0.4 ppm), indicating six degrees of unsaturation. The UV/vis (DAD) spectrum was identical to that of AHBA74, anticipating a homologous chemical structure containing one additional CH_2_ unit, according to the determined molecular formula. The NMR spectra (^1^H, COSY, HSQC, and HMBC) confirmed the expected structural relationship and showed the same pattern of signals for interconverting species at equilibrium. Analysis of the NMR information, especially key COSY and HMBC correlations (Figure 6), and comparison with the NMR spectra of AHBA74 revealed that in this case the molecules are identical by just replacing the methyl ketone in AHB74 by an ethyl ketone in AHB76. Regarding the equilibrating hemiketals, a stereospecific assignment of the signals of each epimer was not carried out.

AHB77 was assigned the molecular formula C_17_H_12_N_2_O_4_ based on the observed [M + H]^+^ ion at *m*/*z* = 309.0875 (calcd. for C_17_H_13_N_2_O_4_^+^ = 309.0870, Δ = 1.6 ppm), indicating thirteen degrees of unsaturation. Its UV/vis (DAD) spectrum suggested the presence of conjugated π systems. A search of the molecular formula in the Dictionary of Natural Products [27] retrieved 6 possible candidates which could account for the UV/vis spectrum obtained. As none of them contained a 3,4-AHBA substructural moiety, 1D ^1^H and 2D NMR spectra (including COSY, NOESY, HSQC, and HMBC) were acquired to determine the structure of the purified compound. Analysis of the ^1^H and HSQC NMR spectra indicated the target compound contains three exchangeable hydrogens. The HSQC spectrum revealed the presence of nine methine groups, one aliphatic and eight of aromatic nature. Detailed analysis of COSY and HMBC key correlations (Figure 6) determined the compound’s connectivity, showing on the one hand the presence of one aromatic (benzene) ring with a 1,3,4 substitution pattern that turned out to correspond to the expected 3,4-AHBA substructural moiety, and on the other hand an indole unit moiety which is connected to an imine carbon contained in a heterocyclic ring fused to the 3,4-AHBA moiety. Such heterocycle is closed via a hemiacetal (according to a δ_C_ of 85.7 for C-8), resembling the ketal closure found in AHB74 and AHB76. Key NOESY correlations further corroborated the established connectivity. The stereochemistry of the hemiacetal chiral center remained unassigned. 

AHB118 was assigned the molecular formula C_10_H_11_NO_5_ based on the observed [M + H]^+^ ion at *m*/*z* = 226.0707 (calcd. for C_12_H_12_NO_5_^+^ = 226.0710, Δ = 1.3 ppm), indicating six degrees of unsaturation. The UV/vis (DAD) spectrum was identical to those of AHB74 and AHB76, anticipating a closely related chemical structure. None of the ten compounds listed in the Dictionary of Natural Products [27] with such a molecular formula contain a 3,4-AHBA substructural moiety. A set of NMR spectra (^1^H, COSY, HSQC, and HMBC) was acquired to elucidate the structure of the purified compound. Interestingly, the ^1^H and HSQC revealed two sets of signals, very close in resonance frequency. Detailed analysis of the NMR spectra confirmed it. To determine the structure of the equilibrating species, the elucidation started from each of the aliphatic methine observed in the region at *δ*_H_ 5.25–5.46, δ_C_ 89.05–91.0 ppm (C-1′). Their chemical shift indicated that such methine signals belong to each of two epimeric hemiacetals in equilibrium, analogous to the epimeric hemiketals found for AHB74 and AHB76. As expected, the determined connectivity of the hemiacetals contains a 3,4-AHBA moiety. Interestingly, in this case the signals of the open aldehyde from which those hemiacetals would be originated are not detected, indicating an equilibrium completely shifted towards the hemiacetalic forms. COSY and HMBC correlations (Figure 6) additionally confirmed the fragment C-1′/C-2′/C-3′ and the linkage of C-1′ to C4 via an oxygen and of C-2′ to C-3 via a nitrogen bridge. The stereochemistry remained undetermined and a stereospecific assignment of the signals of each epimer was not carried out. 

AHB119 was assigned the molecular formula C_9_H_9_NO_4_ based on the observed [M + H]^+^ ion at *m*/*z* = 196.0604 (calcd. for C_9_H_10_NO_4_^+^ = 196.0604, Δ = 0 ppm), indicating six degrees of unsaturation. Its UV/vis (DAD) spectrum suggested the presence of an aromatic or conjugated π system. The molecular formula matches that of an acetylated derivative of 3,4-AHBA. To determine the structure a set of NMR spectra (^1^H, COSY, HSQC, and HMBC) was acquired. The ^1^H and HSQC spectra revealed the presence in the molecule of three sp2 methines and one methyl group with the characteristic chemical shift of an acetyl group. Straightforward analysis of the key COSY and HMBC correlations (Figure 6) rendered the structure of the compound, which corresponded to the *N*-acetyl derivative of 3,4-AHBA. 

AHB120 was assigned the molecular formula C_10_H_11_NO_4_ based on the observed [M + H]^+^ ion at *m*/*z* = 210.0760 (calcd. for C_10_H_12_NO_4_^+^ = 210.0761, Δ = 0.5 ppm), indicating six degrees of unsaturation. Its UV/vis (DAD) spectrum was identical to that of AHB119 and the molecular formula also suggested a possible homologous derivative of AHB119. Analysis of the set of NMR spectra (^1^H, COSY, HSQC, and HMBC) acquired revealed identical aromatic signals, corresponding to the 3,4-AHBA moiety. The key COSY and HMBC correlations (Figure 6) indicated that the compound corresponds to the *N*-propionyl derivative of 3,4-AHBA.

The structure of all AHBs confirms that they all derive from 3,4-AHBA. This is a building block present in different metabolites such as grixazone, bagremycin and ferroverdin, the manumycin family of compounds, cremeomycin, or platensimycin and platencin [30,31,32,33,34,35]. Many of these compounds show antibiotic and antitumour activities, such as platensimycin and platencin that are inhibitors of bacterial type II fatty acid synthases [35]. The antibiotic activity of all AHB compounds was tested by bioassay. Among all compounds, only AHB75 (also known as carboxyexfoliazone or umicyn A) showed some antibiotic activity.

## 3. Discussion

Identification of BGCs in *Streptomyces* genomes is mostly performed using the antiSMASH bioinformatics tool [6,7,8]. However, this and other bioinformatics tools have been developed based on current knowledge of BGCs, which hampers the discovery of novel families of BGCs [36]. In this work, we have used an alternative approach to identify a novel BGC in *S. argillaceus*, consisting in seeking SARP encoding genes in its genome and analyzing their surrounding DNA region. In this way, we have been able to identify the new *ahb* BGC, which had not been retrieved using antiSMASH. This strategy could be used as an additional tool to uncover unknown BGCs. The *ahb* cluster is silent under standard laboratory culture conditions, and is highly preserved in several *Streptomyces* strains, which suggests that their encoding compounds may play an important role when microbial strains face specific environmental conditions. Joint overexpression of three *ahb* regulatory genes (*ahbR2*, *ahbR3,* and *ahbR4*) allowed the activation of the *ahb* cluster and the identification of its encoded AHBs products. We have been able to purify and characterize the chemical structure of seven AHBs, all of them derived from 3,4-AHBA, including four new bicyclic compounds (AHB74, AHB76, AHB77, and AHB118); two acylated 3,4-AHBA (AHB119, also known as 3,4-AcAHBA [24]; and the new AHB120); and the phenoxazinone AHB75 (acetyl-APOC), also known as carboxyexfoliazone [28] or umycin A [29]. Moreover, by overexpressing three genes (*ahbH*, *ahbI,* and *ahbL2*) for the synthesis and activation of 3,4-AHBA, a new hybrid compound, AHB18, was identified, containing a 3,4-AHBA moiety and an ARP bicyclic derivative constituted by a 2,3-dihydropiridine ring fused with a five-membered ring. This ARP moiety closely resembles those synthesized during the final steps of the ARP biosynthetic pathway [17,25,26]. Biosynthesis of AHB18 would result from a metabolic crosstalk between the AHB and the ARP pathways.

As mentioned above, 3,4-AHBA is a building block present in different metabolites such as grixazone [24,34]. The biosynthetic pathway of the phenoxazinone grixazone A starts with the formation of 3,4-AHBA from two metabolites, aspartate semialdehyde (ASA) and dihydroxyacetone-P (DHAP), by an initial aldol condensation catalyzed by GriI to give rise to the acyclic 2-amino-4,5-dihydro-6-one-heptanoate-7-P, followed by a cyclodehydrative aromatization catalyzed by GriH [24]. Formation of 3,4-AHBA is followed by its conversion into an acyl-AMP intermediate by action of the AMP-binding protein GriC, and its reduction to 3-amino-4-hydroxybenzaldehide (3,4-AHBAL) mediated by GriD [37]. Afterwards, formation of the phenoxazinone backbone is preceded by the oxidation of 3,4-AHBAL to its quinone imine by the action of the phenoxazinone synthase GriF [38]. The AHBs biosynthetic pathway would follow similar initial steps as grixazone. The *ahb* cluster contains two genes, *ahbI* and *ahbH*, encoding enzymes with high similarity to fructose-biphosphate aldolases and 3-dehydroquinate synthases II, which would catalyze the initial steps leading to 3,4-AHBA (Figure 2). This would be adenylated by an AMP-binding protein. The *ahb* BGC encodes two acyl-CoA ligases (*ahbL1* and *ahbL2*) that could be involved in adenylation of 3,4-AHBA. Since the 5′-end of *ahbL2* overlaps with the 3′-end of *ahbH*, we propose AhbL2 for this role. As expected, the *ahb* cluster does not encode any homologous to GriD, since AHBs retain the carboxylic group in their molecules. Formation of bicyclic AHBs and the phenoxazinone AHB75 most likely would require the previous oxidation of 3,4-AHBA to its quinone imine, which would be coupled to another quinone imine unit to generate APOC, or to different carbon chains or an indole unit to generate the bicyclic AHBs (Figure 2). In the grixazone and in actinomycin biosynthesis pathways, the oxidation of the corresponding *o*-aminophenols is carried out by phenoxazinone synthases [38,39,40]. The *ahb* cluster lacks any gene encoding that type of enzyme. However, it contains several oxidoreductase coding genes that could be involved in this event. The existence of another acyl-CoA ligase AhbL1 and two kinases (AhbP1 and AhbP2) suggests that condensation of 3,4-AHBA to the different carbon chain and indole units might occur through adenylated or phosphorylated derivatives. Additionally, formation of APOC (precursor of AHB78) could also occur non-enzymatically, although probably more slowly, as it has been reported [38]. In addition, the cluster contains other genes such as methyltransferases, oxygenases, and oxidoreductases that could participate in the biosynthesis of the aliphatic side chains. Further studies will be required to determine the origin of such carbon chains and indole moiety and how they are incorporated to the quinone imine to generate the bicyclic AHBs. AHB119 and AHB120 would be shunt products of the pathway generated by incorporation of acyl groups. Additionally, in the case of AHB75, acylation would be the final steps in its biosynthesis. These acylations would involve acyltransferases. The *ahb* cluster does not contain any acyltransferase coding gene, which indicates that this gene would be in another region of the chromosome. Further studies would be required to clarify the biosynthetic steps downstream of 3,4-AHBA formation. 

Although the reported AHBs did not show antibiotic activity (except AHB75), the identification of the *ahb* genes opens the possibility to use those genes as tools to generate potentially new bioactive compounds by combinatorial biosynthesis. In this sense, we have recently reported a new hybrid antibiotic compound generated by combining genes from the *arp* and *cpk* clusters, which encode the non-bioactive compounds ARP and coelimycin P, respectively [26].

## 4. Materials and Methods

### 4.1. Strains, Culture Conditions, Plasmids, and DNA Manipulations 

*S. argillaceus* ATCC 12956 was used as the source of DNA to express *ahb* genes and to generate mutants in the *ahb* BGC. *Escherichia coli* DH10B (Invitrogen) and *E. coli* ET12567/pUB307 [41] were used as cloning hosts for plasmid propagation and for conjugation experiments, respectively. MA, R5A, and SM10 media [17,42] were used for sporulation or AHB production. When required, antibiotics were added to culture media at the following final concentrations: kanamycin (50 µg/mL), nalidixic acid (25 µg/mL), apramycin (25 µg/mL), hygromycin (200 μg/mL), and thiostrepton (50 µg/mL). Plasmid pCR-Blunt (Invitrogen) and pUO9090 (M. C. Martín, unpublished results) were used for subcloning. Plasmid pHZ1358 [43] was used to generate mutant strains by gene replacement. Plasmids pEM4T [44], pEM4 [45] and pSETe [46] were used for gene expression. pLHyg [47] was used as a source of the hygromycin resistance cassette. DNA manipulations, intergeneric conjugations, and transformations were carried out according to standard procedures for *Streptomyces* [41] and for *E. coli* [48]. PCR amplifications were carried out using Herculase II (Stratagene) and 5% dimethyl-sulfoxide (DMSO). Purified amplicons were sequenced and compared to others in databases. Curated Blast software [21], BLAST [22] and, antiSMASH 6.0 [7] were used for sequence analyses. The maximum-likelihood tree was generated by using the autoMLST server [49]. An IQ-TREE Ultrafast Bootstrap analysis (1000 replicates) was performed, and ModelFinder was applied to find the optimal model for tree building. Genbank files containing the genomic sequences from the strains under study were used as inputs for phylogenetic inference using “de novo mode” pipeline, as in Ceniceros et al. [23].

### 4.2. Plasmid Constructs to Generate Mutant Strains 

Several plasmids were constructed to generate knock out mutant strains (Table 1), using oligoprimers from Table S3:

pHZΔasu: This plasmid was used for jointly deleting *ahbH* and *ahbI*. First, a 2.11 kb DNA fragment containing the 5′-end of *ahbR4*, *ahbL2* and the 3′-end of *ahbH* was amplified using oligonucleotides Delta ASU A fwd and Delta ASU A rev, digested with EcoRI and PstI and cloned upstream the apramycin resistance cassette in pUO9090 digested with the same enzymes. Secondly, a 2 kb DNA fragment containing the 5′-end of *ahbI*, *ahbK5* and the 5′-end of *ahbO4* was amplified using oligonucleotides Delta ASU B fwd and Delta ASU B rev, digested with BamHI and EcoRV and cloned downstream the apramycin resistance cassette in the above pUO9090-derivative generated plasmid that was digested with the same enzymes. Finally, the whole insert in pUO9090 was recovered as a SpeI fragment and cloned into the XbaI site of pHZ1358, generating pHZΔasu.
pHZΔasu1705: This plasmid was used for deleting a DNA region from *ahbR1* to *ahbK3*. First, a 1.9 kb DNA fragment containing the 5′-end of *ahbO1*, *ahbM1*, *ahbM2* and the 3′-end of *ahbR1* was amplified using oligonucleotides Delta 1705A fwd and Delta 1705A rev, digested with EcoRI and PstI (this last cutting within *ahbR1*) and cloned upstream the apramycin resistance cassette in pUO9090 digested with the same enzymes. Additionally, a 2 kb DNA fragment containing the 3′-end of *ahbK3*, *ahbM4*, *ahbK4* and the 5′-end of *ahbO3* was amplified using oligonucleotides Delta 1705B fwd and Delta 1705B rev, digested with BamHI and EcoRV and cloned downstream the apramycin resistance cassette in the pUO9090 plasmid containing the first fragment and digested with the same enzymes. Finally, the whole fragment was released as a SpeI fragment and cloned into the XbaI site of pHZ1358, generating pHZΔasu1705.

These plasmids were introduced into *S. argillaceus* to generate *S. argillaceus* ΔAHBA and *S. argillaceus* ΔR2K3 mutant strains. Mutants were selected by their apramycin-resistance and thiostrepton-sensitive phenotype, and their genotypes were further verified by PCR amplification with appropriate oligonucleotides (Table S2; Figures S2 and S3) and by sequencing the resultant PCR products. 

### 4.3. Plasmid Constructs for Gene Expression

Several plasmids were constructed to express several *ahb* genes (Table 1), using oligoprimers from Table S3 as follows:

pEM4T-AHBA: This plasmid was used to overexpress *ahbI*, *ahbH,* and *ahbL2*. These genes were amplified as a 3.84 kb fragment using oligonucleotides AHBAermE fwd and AHBAermE rev, digested with BamHI and EcoRI, and cloned downstream of the erythromycin resistance promoter in pEM4T digested with the same enzymes. The final plasmid pEM4T-AHBA was introduced into *S. argillaceus* WT to generate *S. argillaceus* WT-pEM4T-AHBA strain.
pREGT: This plasmid was used for overexpressing *ahbR2*, *ahbR3,* and *ahbR4*. First, *ahbR2* was amplified as a 0.92 kb fragment using oligonucleotides SARP1304 fwd and SARP1304 rev, cloned into pCR-Blunt, recovered as a PstI fragment, and cloned into the PstI site of pEM4 downstream of the erythromycin resistance promoter, generating pEM4-ahbR2. Second, *ahbR3* was amplified as a 1 kb fragment using oligonucleotides SARP1705 fwd and SARP1705 rev, cloned into pCR-Blunt, recovered as a XbaI fragment, and cloned into the XbaI site, in the right orientation, downstream of pEM4-ahbR2 to generate pEM4-ahbR2R3. Third, *ahbR4* was amplified as a 1.26 kb fragment using oligonucleotides 1705araC fwd and 1705araC rev, cloned into pCR-Blunt, recovered as an EcoRI fragment, and cloned in the right orientation, in the EcoRI site of the downstream of *ahbR3* in pEM4-ahbR2R3, generating pREG. Finally, the *oriT* fragment was recovered from pEM4T as a PstI fragment, cloned into pCR-Blunt, recovered as a HindIII fragment, and cloned into the same site of the pREG. The final plasmid pREGT was introduced into *S. argillaceus* WT, ΔAHBA and ΔR2K3 to generate *S. argillaceus* WT-pREGT, ΔAHBA-pREGT and ΔR2K3-pREGT, respectively.
pSETeAHBAHyg: This plasmid was used to complement *S. argillaceus* ΔAHBA. Genes *ahbI*, *ahbH,* and *ahbL2* were amplified as a 3.84 kb fragment using oligonucleotides AHBAermE fwd and AHBAermE rev, cloned into pCR-Blunt, and recovered as a BamHI-EcoRI fragment to be cloned in the right orientation downstream of the erythromycin resistance promoter in pSETe digested with the same enzymes, to generate pSETeAHBA. Then, a hygromycin resistance cassette was obtained from pLHyg as an EcoRV fragment and cloned in pSETeAHBA digested with NheI and filled ends with Klenow. The final plasmid pSETeAHBAHyg was introduced into *S. argillaceus* ΔAHBA-pREGT to generate *S. argillaceus* ΔAHBA-pREGT-pSETeAHBAHyg strain.

### 4.4. Feeding Experiments

Feeding experiments were carried out in 24-square deep-well plates, containing 3 mL of R5A medium and 3,4-AHBA (100 µM final concentration). After 24 h of incubation, samples of 1 mL were harvested for further extraction with ethyl acetate or *n*-butanol. 

### 4.5. Extraction, UPLC Analysis and Purification of Ahbamycins

AHBs were extracted from cultures with equal volumes of either *n*-butanol (AHB18) or ethyl acetate containing 1% formic acid (AHB74 to AHB77, and AHB118 to AHB120). Preliminary analyses were carried out by reversed-phase chromatography, as previously reported [17]. Detection and UV-based identification was performed by photodiode array detection. Chromatograms were extracted at 230 nm, 360 nm, and 400 nm. For purification purposes, *S. argillaceus* strains were grown by a two-step culture method, as previously described [42], using forty 250-mL Erlenmeyer flasks in the production step. AHB18 was purified from 7 days old cultures of *S. argillaceus* WT-pEM4T-AHBA in R5A, while AHB74 to AHB747 and AHB118 to AHB120 were purified from cultures of *S. argillaceus* WT-pREGT in SM10. Purification of AHB18 and AHB74 to AHB77 was carried out, as previously described [17], using isocratic chromatography conditions optimized for each compound. In the case of AHB118 to AHB120, as they were not retained in the solid-phase extraction cartridge, they were recovered from the non-retained material by ethyl acetate extraction, followed by evaporation *in vacuo*, and purification by preparative HPLC. 

### 4.6. Structural Elucidation of Ahbamycins 

Structural elucidation of the new compounds was carried out using a combination of ESI-TOF mass spectrometry and NMR spectroscopy (see Supplementary Materials). HRMS spectra were collected from LC-DAD-MS analyses using an Agilent 1200 Rapid Resolution HPLC system equipped with a SB-C8 column (2.1 × 30 mm, Zorbax) and coupled to a Bruker maXis mass spectrometer. Chromatographic and ionization conditions were identical to those previously described [50,51]. UV/vis (DAD) spectra were also collected in the same chromatographic analyses. NMR spectra were recorded in CD_3_OD or DMSO-d6 at 24 °C on a Bruker AVANCE III-500 (500 MHz and 125 MHz for 1H and 13C NMR, respectively) equipped with a 1.7 mm TCI MicroCryoProbeTM, using the residual solvent signal as internal reference (δ_H_ 3.31 and δ_C_ 49.0 for CD_3_OD; δ_H_ 2.51 and δ_C_ 40.0 for DMSO-d_6_). The molecular formula obtained from the experimental accurate mass of each compound combined with the analysis of the 1D and 2D NMR spectra rendered the chemical structure of the compounds.

### 4.7. Accession Codes

The sequence of *Streptomyces argillaceus ahb* gene cluster has been deposited at GenBank under the accession number OQ117053.

## Figures and Tables

**Table 1:** Strains and plasmids generated in this study.

Mutant Strain	Plasmid	Deleted Genes
ΔAHBA	pHZΔasu	*ahbH*, *ahbI*
ΔR2K3	pHZΔasu1705	*ahbR1*, *ahbR2*, *ahbP1*, *ahbA*, *ahbS*, *ahbK1*, *ahbM3*, *ahbL1*, *ahbO2*, *ahbR3*, *ahbT1*, *ahbK2*, *ahbC*, *ahbK3*
**Recombinant Strain**	**Plasmid**	**Expressed Genes**
WT-pEM4T	pEM4T	-
WT-pEM4T-AHBA	pEM4T-AHBA	*ahbI*, *ahbH*, *ahbL2*
WT-pREGT	pREGT	*ahbR2*, *ahbR3*, *ahbR4*
ΔAHBA-pREGT	pREGT	*ahbR2*, *ahbR3*, *ahbR4*
ΔR2K3-pREGT	pREGT	*ahbR2*, *ahbR3*, *ahbR4*
ΔAHBA-pREG-pSETeAHBAHyg	pREGT pSETeAHBAHyg	*ahbR2*, *ahbR3*, *ahbR4* *ahbI*, *ahbH*, *ahbL2*

## Data Availability

The data presented in this study are available in the article and in Supplementary Materials.

## Supplementary Materials

The following supporting information can be downloaded at: https://www.mdpi.com/article/10.3390/ijms24098197/s1.
