# Supplementary material for: Uncovering the Cryptic Gene Cluster ahb for 3-amino-4-hydroxybenzoate Derived Ahbamycins, by Searching SARP Regulator Encoding Genes in the Streptomyces argillaceus Genome
Source: Int J Mol Sci. 2023 May 3;24(9):8197. doi: 10.3390/ijms24098197 (PMC10179220; doi:10.3390/ijms24098197)
Supplement: Supplementary file 1 [file ijms-24-08197-s001.zip › ijms-2345644-supplementary.pdf]

SUPPLEMENTARY MATERIAL

Uncovering the cryptic gene cluster *ahb* for 3-amino-4-hydroxybenzoate derived ahbamycins, by searching SARP regulator encoding genes in the *Streptomyces argillaceus* genome

Suhui Ye,[†,‡] Brian Molloy,[†] Ignacio Pérez-Victoria,[#] Ignacio Montero,[†,‡] Alfredo F. Braña,[†] Carlos Olano,[†,‡] Sonia Arca,[†] , Jesús Martín,[#] Fernando Reyes,[#] José A. Salas[†,‡] and Carmen Méndez[†,‡] *

[†]Departamento de Biología Funcional e Instituto Universitario de Oncología del Principado de Asturias (I.U.O.P.A), Universidad de Oviedo, Oviedo, Spain

[‡]Instituto de Investigación Sanitaria de Asturias (ISPA), Oviedo, Spain

[#]Fundación MEDINA, Centro de Excelencia en Investigación de Medicamentos Innovadores en Andalucía, Armilla, Granada, Spain.

* Correspondence: Carmen Méndez (cmendezf@uniovi.es)

Table S1. Secondary metabolite gene clusters identified in *S. argillaceus* using antiSMASH 6.0

Cluster	Type	From	to	Predicted product
1	Butyrolactone	128,191	136,902	Unknown
2	Thiopeptide, PKS-like	171,777	225,841	Lactazole
3	Lanthipeptide-class III	401,954	424,038	SapB
4	NRPS-like	560,900	599,983	Unknown
5	Type II PKS, oligosaccharide	819,530	891,026	Mithramycin [18]
6	Lassopeptide, RiPP-like	1,034,574	1,056,293	Citrulassin D
7	Type II PKS	1,062,634	1,135,149	Spore pigment
8	NAPAA	1,638,191	1,672,045	ε-Poly-L-Lysine
9	Ectoine	1,875,607	1,886,011	Ectoine
10	TransAT-PKS-like, NRPS-like	2,346,931	2,423,228	Largimycins [20]
11	Melanin	2,866,180	2,875,776	Melanin
12	NI-Siderophore	2,971,449	2,982,026	Desferrioxamine B [19]
13	NRPS	3,407,302	3,461,180	Unknown
14	Terpene	5,232,759	5,251,815	Albaflavenone
15	Lanthipeptide-class II	5,316,978	5,340,023	Unknown
16	Siderophore	5,836,456	5,847,118	Unknown
17	Type I PKS	6,055,193	6,122,436	Argimycins P [17]
18	RiPP-like	6,309,087	6,319,089	Unknown
19	Terpene, butyrolactone	6,346,491	6,367,657	γ-butyrolactone
20	NI-Siderophore	6,488,758	6,501,948	Unknown
21	Indole	6,700,660	6,721,835	Unknown
22	Terpene	6,916,343	6,942,645	Hopene
23	hglE-KS-Type I PKS	6,991,030	7,043,896	Unknown
24	RiPP-like	7,270,946	7,281,161	Unknown
25	Melanin	7,430,918	7,441,352	Unknown
26	Terpene	7,564,422	7,590,728	Isorenieratene [19]
27	NRPS, Type I PKS	7,905,486	7,954,919	Antimycin [19]
28	Thioamide-NRP, NRPS, lanthipeptide-class II	8,021,022	8,112,102	Unknown
29	Lantipeptide-class I	8,224,262	8,249,451	Unknown
30	Lassopeptide	8,450,703	8,473,371	Unknown
31	NRPS	8,614,230	8,667,317	Unknown

hglE-KS, Heterocyst glycolipid synthase-like PKS; NI-siderpophore, NRPS-independent; NAPAA, Non-alpha poly-amino acids like e-Polylysin; NRPS, Non-Ribosomal Peptide Synthetase; PKS, Polyketide Synthase; RiPP, Ribosomally synthesized and post-translationally modified peptide.

Table S2. Predicted functions of genes in the *Streptomyces argillaceus ahb* gene cluster

ahb*	**aa**	**Predicted function**	***rah****	***oah****	***dah****	***pah****	***tah****
ahbO1	281	2OG-Fe(II) oxygenase	WP_217236194 (94)	WP_189953973 (92)	WP_202919556 (92)	WP_079170665 (91)	WP_159539127 (89)
ahbM1	182	Methylated-DNA-[protein]-cysteine S-methyltransferase	WP_217236059 (89)	WP_189953954 (84)	WP_152883716 (89)	WP_055572750 (87)	WP_159536550 (88)
ahbM2	254	MGMT family protein	WP_217236195 (89)	WP_189953956 (89)	WP_152883945 (91)	WP_095534003 (86)	WP_159536551 (84)
ahbR1	197	RNA polymerase sigma factor	WP_217236060 (94)	WP_189953958 (93)	WP_152883713 (95)	WP_055572748 (93)	WP_159536552 (88)
ahbR2	258	AfsR/SARP family transcriptional regulator	WP_217236061 (91)	WP_189953960 (87)	WP_152883711 (93)	WP_055572747 (89)	WP_159536553 (87)
ahbP1	322	PfkB family carbohydrate kinase	WP_217236062 (90)	---	WP_152883709 (90)	---	WP_159536554 (89)
ahbA	406	Methionine adenosyltransferase	WP_217236063 (93)	WP_189948684 (93)	WP_152883707 (95)	WP_055572746 (92)	WP_159536555 (92)
ahbS	469	3-carboxy-cis,cis-muconate cycloisomerase	WP_217236064 (92)	WP_189948686 (89)	---	WP_055572745 (92)	WP_159536556 (86)
ahbK1	656	FAD/NAD(P)-binding protein	WP_217236065 (89)	WP_189948687 (89)	WP_152883705 (89)	WP_055572744 (86)	WP_159536557 (87)
ahbM3	337	Methyltransferase	WP_217236066 (94)	WP_189948689 (94)	WP_152883703 (94)	WP_055572743 (92)	WP_159536558 (94)
ahbL1	572	Acyl-CoA ligase	WP_217236067 (91)	WP_189948692 (92)	WP_152883700 (94)	WP_055572742 (91)	WP_159536559 (89)
ahbO2	584	FAD-dependent monooxygenase	WP_217236068 (90)	WP_189948694 (91)	WP_152883698 (92)	WP_055572741 (92)	WP_006124753 (ND)
ahbR3	290	AfsR/SARP family transcriptional regulator	WP_217236069 (93)	WP_229851348 (88)	WP_152883695 (90)	WP_074993823 (90)	WP_159536560 (80)
ahbT1	536	MFS transporter	WP_217236070 (86)	WP_189948698 (87)	WP_152892632 (91)	SDC40704.1 (89)	WP_159536561 (78)
ahbK2	268	SDR family oxidoreductase	WP_217236071 (99)	WP_189948700 (97)	WP_152892631 (98)	WP_055574900 (97)	WP_159536562 (99)
ahbC	208	Cupin domain-containing protein	WP_217236072 (97)	WP_229851349 (96)	WP_152892630 (98)	WP_074993828 (96)	WP_159536563 (96)
ahbK3	566	FAD-binding oxidoreductase	WP_217236073 (89)	WP_229851350 (90)	WP_152892629 (89)	WP_079039606 (90)	WP_159536564 (86)
ahbM4	220	Class I SAM-dependent methyltransferase	WP_217236074 (95)	WP_189948706 (95)	WP_152892628 (96)	WP_055570494 (95)	WP_159536565 (96)
ahbK4	188	Flavin reductase family protein	WP_217236075 (93)	WP_010070850 (ND)	WP_152892627 (92)	WP_055570493 (93)	WP_159536566 (89)
ahbO3	550	Aromatic ring hydroxylase	WP_217236076 (98)	WP_189948710 (98)	WP_152892626 (97)	WP_055570492 (99)	WP_159536567 (97)
ahbT2	405	MFS transporter	WP_217236077 (93)	WP_189948712 (91)	WP_152892625 (93)	WP_074993830 (93)	WP_159536568 (90)
ahbR4	305	Helix-turn-helix domain-containing protein	WP_217236078 (91)	WP_189948715 (87)	WP_152892624 (93)	WP_074993831 (88)	WP_159536569 (84)
ahbL2	584	Acyl-CoA ligase	WP_217236079 (86)	WP_189948717 (84)	WP_152892623 (90)	WP_055574594 (87)	WP_006124740 (ND)
ahbH	369	3-dehydroquinate synthase II family protein	WP_217236080 (97)	WP_189948720 (95)	WP_006124740 (ND)	WP_074993834 (97)	WP_159536570 (94)
ahbI	271	Fructose-bisphosphate aldolase	WP_217236196 (94)	WP_189948963 (97)	WP_152892636 (94)	WP_079039969 (95)	WP_159539129 (94)
ahbK5	550	FAD-binding oxidoreductase	WP_217236081 (92)	WP_229851351 (90)	WP_152892622 (93)	WP_095534094 (94)	WP_208026539 (90)
ahbO4	502	FAD-dependent monooxygenase	WP_217236082 (93)	WP_189948723 (90)	WP_152892621 (92)	WP_055694972 (94)	WP_159536571 (92)
ahbF	299	Methylenetetrahydrofolate reductase	WP_217236083 (91)	WP_189948725 (90)	WP_152892620 (89)	WP_055572786 (90)	WP_159536572 (89)
ahbP2	327	Carbohydrate kinase family protein	WP_217236084 (90)	WP_189948727 (89)	WP_152892635 (91)	WP_055572784 (91)	WP_159536574 (89)
ahbR5	100	Metal-binding protein	WP_217236197 (85)	WP_189948729 (80)	WP_202919764 (82)	WP_079039791 (86)	---
ahbT3	173	ABC transporter substrate-binding protein	WP_217236085 (87)	WP_229851352 (82)	WP_152892619 (84)	WP_055572783 (87)	---

* *ahb* ,*S. argillaceus* ATCC 12956*; rah*, *Streptomyces* sp. AC555_RSS877; *oah*, *S. roseolus* JCM 4411; *dah*, *S. adustus* NBRC 109810; *pah*, *S. prasinopilosus* CGMCC 4.3504; *tah*, *Streptomyces* sp. Tü 3180. Numbers between brackets indicate percentage of identical amino acids to the corresponded Ahb protein. ND, not determined. aa, number of amino acids of Ahb proteins.

Table S3. Oligonucleotides used for PCR amplification (restriction sites are underlined).

PRIMER	SEQUENCE 5'-3'
**Delta ASU A fwd**	GGGGAATTCGAAGAAGCGGACGTTCTCG
**Delta ASU A rev**	CGGCTGCAGATCGTGCAGGACGACTGG
**Delta ASU B fwd**	CGGGGATCCGGACACGGAGTGGTCCAG
**Delta ASU B rev**	TTGGATATCGGTACGTCAGGAGGAACACC
**Delta 1705A fwd**	CACGAATTCCAGGTGAGGGGTGTGGTAAC
**Delta 17054A rev**	CGTTCCTGTACCGGGTCTC
**Delta 1705B fwd**	ACCGGATCCTGCAGCAGGTGAAGAAGAAG
**Delta 1705B rev**	GTTGATATCCGCGTAGTAGTCGGAGTTGG
**AHBAermE fwd**	GGATCCATCCGAACCCTGTGACCAG
**AHBAermE rev**	GAATTCTGACCACCACGTAGCAGAAC
**SARP1304 fwd**	CTGCAGGGCTCTCCTACGCTCTCTCA
**SARP1304 rev**	CTGCAGCTGCCTTGTCGTCCATCAC
**SARP1705 fwd**	TCTAGATTCCAGAGGGATGACCTCCT
**SARP1705 rev**	TCTAGAGTGGGCGCCCTGGTCTGG
**1705araC fwd**	CATCACCTGGTGACCGGCGCGG
**1705araC rev**	CCCGGCTACCGGCTCGGGA
**Check Delta AHBA fwd**	ACCAGGACCGTTGTGTCATC
**Check Delta AHBA rev**	CTGTGACCAGGAACGAGGAG
**d1705compr_A**	AACATGTCGTCCCGGTC
**d1705compr_B**	TTCCAGAACAGCACCTTCT
**ApraC rev**	TCATTCTGTGGGCCGTAC
**ApraC fw**	TCATTCTGTGGGCCGTAC

Table S4. NMR data for **AHB18** (CD_3OD, 24 °C, 500 MHz/125 MHz).

Position	δ_H (ppm), mult. (*J* in Hz)	δ_C (ppm), type
2	3.57, at (7.6)	38.2, CH_2
3	2.72, ddd (7.6, 7.6, 4.5)	22.9, CH_2
4	7.09, at (4.5)	130.9, CH
4a	-	128.4, C
5	-	123.1, C
6	-	168.3, C
7	5.50, s	94.0, CH
7a	-	166.3, C
8	7.22, d (11.1)	129.1, CH
9	6.94, m	126.4, CH
10	6.46, m	142.9, CH
11	2.03, br d (6.8)	17.8, CH_3
1’	-	126.4, C
2’	7.86, br s	127.8, CH
3’	-	124.3, C
4’	-	155.0, C
5’	7.00 d, (8.9)	115.7, CH
6’	7.87, br d	130.4, CH
7’	–	170.5, C

Nitrogen	δ_N (ppm)
1	108
3’ NH_2	110

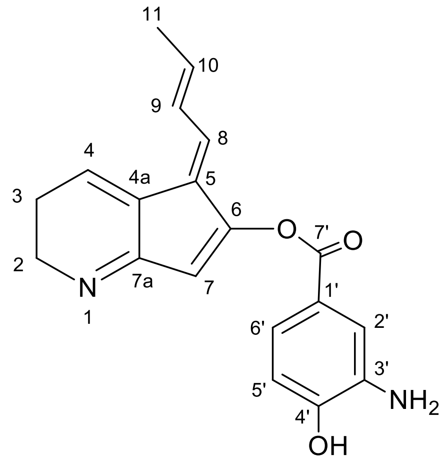

^{13}C chemical shifts determined from the indirect dimension of HSQC and HMBC spectra. ^{15}N chemical shift scale corresponds to Bruker’s default ammonia reference.

Table S5a. NMR data for keto form of **AHB74** (CD_3OD, 24 °C, 500 MHz/125 MHz).

Position	δ_H (ppm), mult. (*J* in Hz)	δ_C (ppm), type
1	-	122.3, C
2	6.96, d (1.9)	111.2, CH
3	-	135.5, C
4	-	149.2, C
5	6.76, d (8.1)	113.5, CH
6	7.18, dd (8.1, 1.9)	120.2, CH
7	-	168.1, C
1’	2.14, s	26.0, CH_3
2’	-	210.5, C
3’	4.12, quart (7.0)	58.1, CH
4’	1.34, d (7.0)	17.4, CH_3

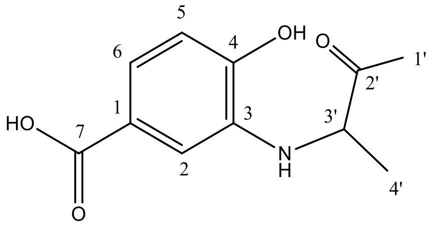

^{13}C chemical shifts determined from the indirect dimension of HSQC and HMBC spectra.

Table S5b. NMR data for hemiketal form of **AHB74** (CD_3OD, 24 °C, 500 MHz/125 MHz). Non isochronous signals of both hemiketal epimers (at position 2') are indicated.

Position	δ_H (ppm), mult. (*J* in Hz)	δ_C (ppm), type
1	-	123.5, C
2	7.23, m	115.9, CH
3	-	133.2, C
4	-	146.4, C
5	6.68, d (8.3)	116.3, CH
6	7.17, m	119.9, CH
7	-	167.9, C
1' (epimer a)<br>1' (epimer b)	1.41, s<br>1.25, s	24.7, CH_3<br>21.3, CH_3
2' (epimer a)<br>2' (epimer b)	-<br>-	96.4, C<br>98.5, C
3' (epimer a)<br>3' (epimer b)	3.14, quart. (6.3)<br>3.13, quart. (6.3)	52.5, CH
4' (epimer a)<br>4' (epimer b)	1.14, d (6.3)<br>1.10, d (6.3)	16.3, CH_3<br>16.7, CH_3

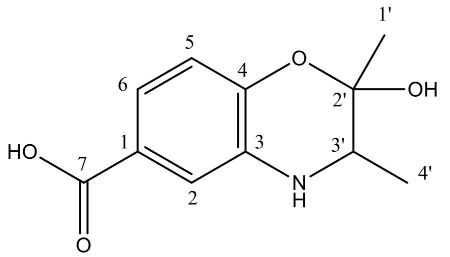

^{13}C chemical shifts determined from the indirect dimension of HSQC and HMBC spectra.

Table S6. NMR data for **AHB75** (DMSO-d_6, 24 °C, 500 MHz/125 MHz).

Position	δ_H (ppm), mult. (*J* in Hz)	δ_C (ppm), type
1a	-	149.1, C
1	8.28, s	113.9, CH
2	-	138.5, C
2-NH	9.77, s	-
3	-	180.2, C
4	6.54, s	104.9, CH
4a	-	150.2, C
5a	-	146.3, C
6	7.63, d (8.6)	117.1, CH
7	8.12, dd (8.6, 2.0)	132.6, CH
8	-	128.6, C
9	8.29, d (2.0)	130.8, CH
9a	-	133.6, C
10	-	166.6, C
11	-	171.3, C
12	2.25, s	24.9, CH

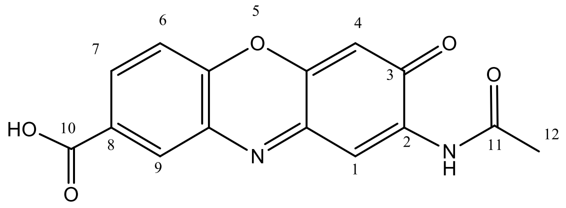

^{13}C chemical shifts determined from the indirect dimension of HSQC and HMBC spectra.

Table S7a. NMR data for the keto form of **AHB76** (DMSO-d_6, 24 °C, 500 MHz/125 MHz).

Position	δ_H (ppm), mult. (*J* in Hz)	δ_C (ppm), type
1	-	122.5, C
2	6.92,d (1.6)	110.9, CH
3	-	135.9, C
4	-	149.0, C
5	6.74, d (8.1)	113.4, CH
6	7.15, dd (8.1, 1.8)	119.8, CH
7	-	168.3, C
1’	0.93, m	8.0, CH_3
2’	2.61, m<br>2.50, m	31.1, CH_2
3’	-	213.2, C
4’	4.14, quart (7.0)	57.4, CH
5’	1.33, d (7.0)	17.8, CH_3

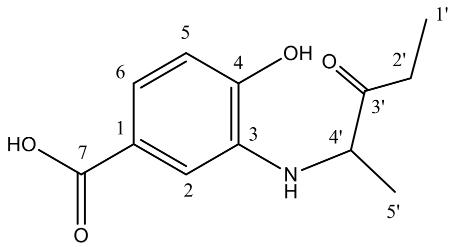

^{13}C chemical shifts determined from the indirect dimension of HSQC and HMBC spectra.

Table S7b. NMR data for the hemiketal form of **AHB76** (DMSO-d_6, 24 °C, 500 MHz/125 MHz). Non isochronous signals of both hemiketal epimers (at position 3’) are indicated.

Position	δ_H (ppm), mult. (*J* in Hz)	δ_C (ppm), type
1	-	123.7, C
2	7.20, d (1.2)	115.7, CH
3	-	134.0, C
4	-	146.2, C
5	6.67, d (8.1)	116.2, CH
6	7.12, m	119.4, CH
7	-	168.1, C
1’ (epimer a)<br>1’ (epimer b)	0.96, t (7.5)<br>0.90, t (7.6	7.6, CH_3<br>7.0, CH_3
2’ (epimer a)<br>2’ (epimer b)	1.77, dq (15.2, 7.5); 1.66, m<br>1.60, m	30.2, CH_2<br>26.9, CH_2
3’ (epimer a)<br>3’ (epimer b)	-<br>-	97.7, C<br>98.8, C
4’ (epimer a)<br>4’ (epimer b)	3.18, quart. (6.5)<br>3.22, m	50.1, CH<br>51.5, CH
5’ (epimer a)<br>5’ (epimer b)	1.10, d (6.5)<br>1.06, d (6.5)	16.1, CH_3<br>16.9, CH_3

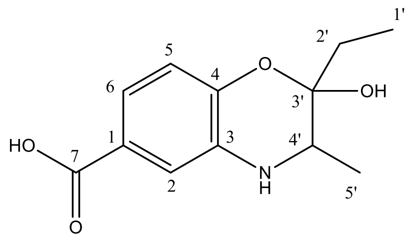

^{13}C chemical shifts determined from the indirect dimension of HSQC and HMBC spectra.

Table S8. NMR data for **AHB77** (DMSO-d_6, 24 °C, 500 MHz/125 MHz).

Position	δ_H (ppm), mult. (J in Hz)	δ_C (ppm), type
1	-	125.2, C
2	7.99, d (2.1)	128.4, CH
3	-	134.0, C
4	-	148.2, C
5	7.10, d (8.3)	117.2, CH
6	7.73, m	128.4, CH
7	-	167.6, C
8	6.42, d (6.7)	85.7, CH
8-OH	7.74, m	-
9	-	156.8, C
11	-	112.5, C
12	8.16, d (2.9)	131.2, CH
13	11.88, br s	-
14	-	137.5, C
15	7.50, br dt (7.6)	112.5, CH
16	7.25, m	123.3, CH
17	7.22, m	121.6, CH
18	8.60, br dd (7.3)	123.1, CH
19	-	125.9, C

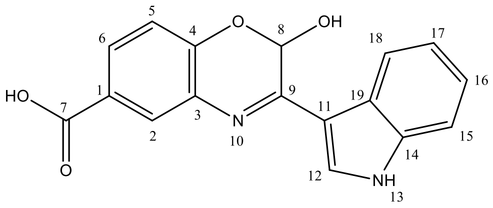

^{13}C chemical shifts determined from the indirect dimension of HSQC and HMBC spectra.

Table S9. NMR data for **AHB118** (DMSO-d_6, 24 °C, 500 MHz/125 MHz). Non isochronous signals of both hemicetal epimers (at position 1') are indicated.

Position	δ_H (ppm), mult. (J in Hz)	δ_C (ppm), type
1	-	123.7, C
2	7.23, d (1.8)	115.5, CH
3	-	134.0, C
4	-	145.2, C
5	6.70, d (8.2)	116.4, CH
6	7.12, dd (8.2, 1.8)	119.0, CH
7	-	168.0, C
1' (epimer a)	5.29, d (3.2)	90.4, CH
1' (epimer b)	5.44, d (1.4)	90.3, CH
2' (epimer a)	3.11, ddd (6.3, 6.3, 3.3)	55.7, CH
2' (epimer b)	3.27, ddd (6.4, 6.4, 1.9)	54.9, CH
3' (epimer a)	3.45, m; 3.35, m	62.0, CH_2
3' (epimer b)	3.57, dd (6.3, 3.8); 3.43, m	61.1, CH_2

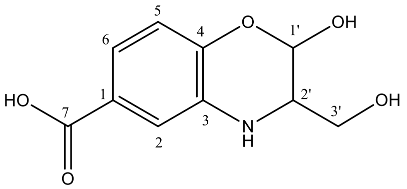

^{13}C chemical shifts determined from the indirect dimension of HSQC and HMBC spectra.

Table S10. NMR data for **AHB119** (DMSO-d_6, 24 °C, 500 MHz/125 MHz).

Position	δ_H (ppm), mult. (*J* in Hz)	δ_C (ppm), type
1	-	121.8, C
2	8.42, d (1.9)	124.1, CH
3	-	126.6, C
3-NH	9.30, s	-
4	-	152.8, C
5	6.92, d (8.3)	115.5, CH
6	7.57, dd (8.3, 1.9)	126.8, CH
7	-	167.6, C
1’	-	169.5, C
2’	2.11, s	24.1, CH_3

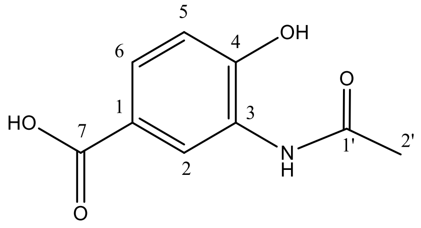

^{13}C chemical shifts determined from the indirect dimension of HSQC and HMBC spectra.

Table S11. NMR data for **AHB120** (DMSO-d_6, 24 °C, 500 MHz/125 MHz).

Position	δ_H (ppm), mult. (*J* in Hz)	δ_C (ppm), type
1	-	121.8, C
2	8.42, d (1.9)	124.1, CH
3	-	126.6, C
3-NH	9.30, s	-
4	-	152.8, C
5	6.92, d (8.3)	115.5, CH
6	7.57, dd (8.3, 1.9)	126.8, CH
7	-	167.6, C
1’	-	173.1, C
2’	2.41, quart. (7.6)	29.7, CH_2
3’	1.08, t (7.6)	10.2, CH_3

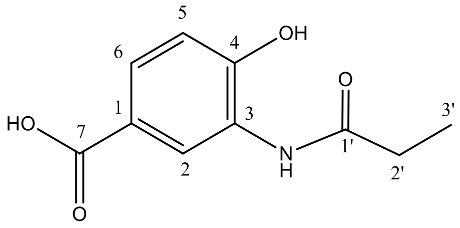

^{13}C chemical shifts determined from the indirect dimension of HSQC and HMBC spectra.

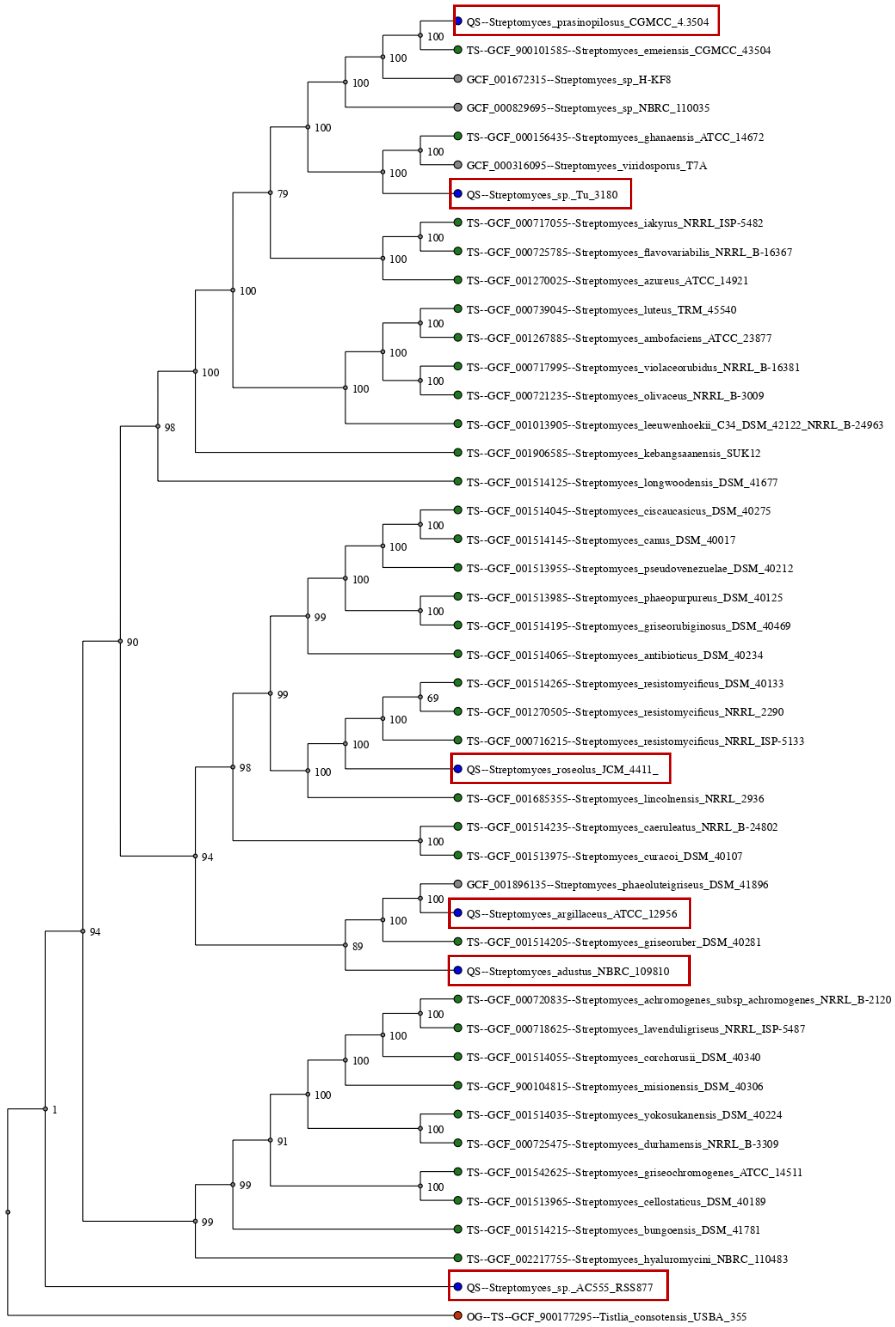

Figure S1. Phylogenetic analysis of *Streptomyces* strains containing *ahb* BGCs. The maximum-likelihood tree of the concatenated nucleotide sequences of 82 housekeeping genes were generated with autoMLST server [49]. An IQ-TREE Ultrafast Bootstrap analysis (1000 replicates) was performed, and ModelFinder was applied to find the optimal model for tree building. Genbank files containing the genomic sequences from the strains under study were used as inputs for phylogenetic inference using “de novo mode” pipeline, as in Ceniceros et al. [23]. Strains used for *ahb* clusters comparison are squared in red. TS: Type strain, QS: query sequence, OG: outgroup.

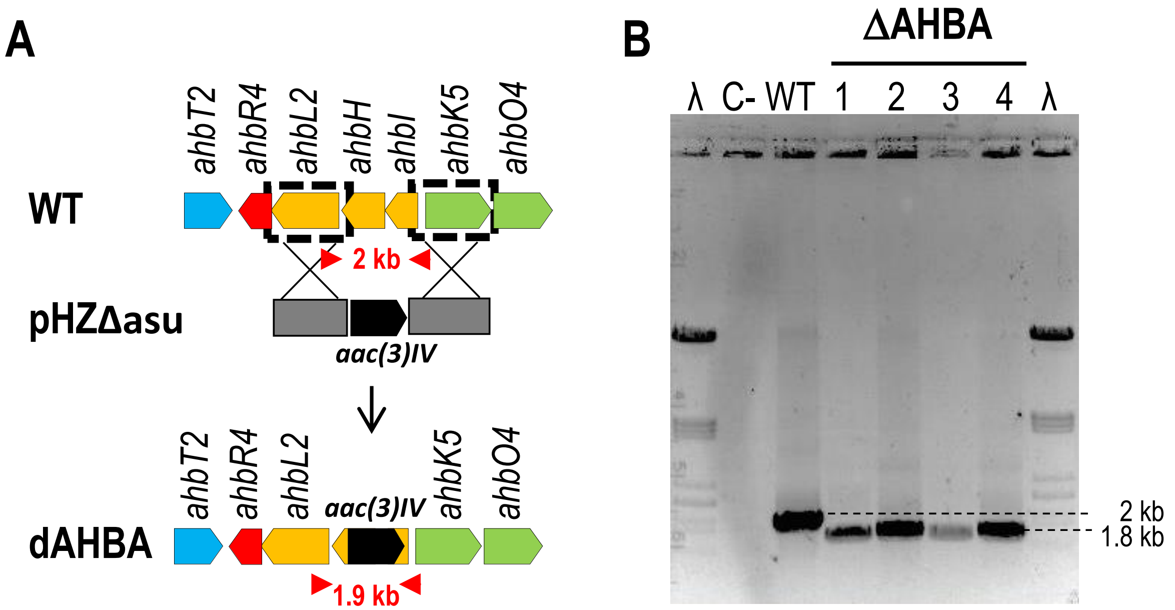

Figure S2. Generation of *S. argillaceus* ΔAHBA. (A) Graphical representation of the replacement event for the generation of ΔAHBA mutant strain; (B) PCR analysis of ΔAHBA mutant strain. PCR products from the wild type (WT) strain and from the ΔAHBA mutant using oligonucleotides Check Delta AHBA fw (a) and Check Delta AHBA rev (b). Negative control of the PCR reaction (-). λ, PstI-digested Lambda DNA. *aac(3)IV*, apramycin resistance gene.

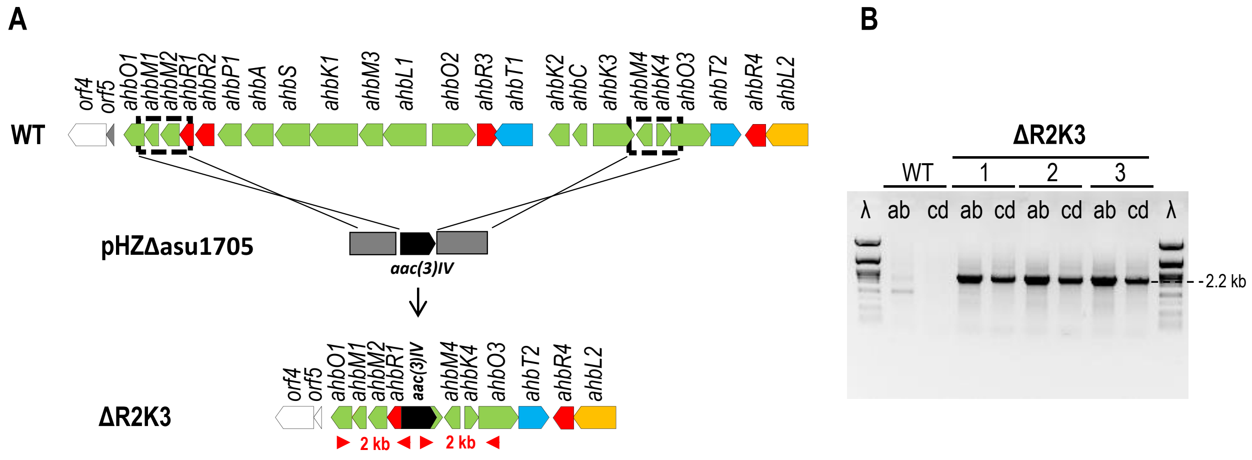

Figure S3. Generation of *S. argillaceus* ΔR2K3. (A) Graphical representation of the replacement event for the generation of ΔR2K3 mutant strain; (B) PCR analysis of ΔR2K3 mutant strain. PCR products from the ΔR2K3 mutant using oligonucleotides d1705compr_A (a) and ApraC rev (b), or d1705compr_B (c) and ApraC fw (d). Oligonucleotides b and c anneals at the apramycin resistance gene. WT was used as negative control. λ, PstI-digested Lambda DNA. *aac(3)IV*, apramycin resistance gene

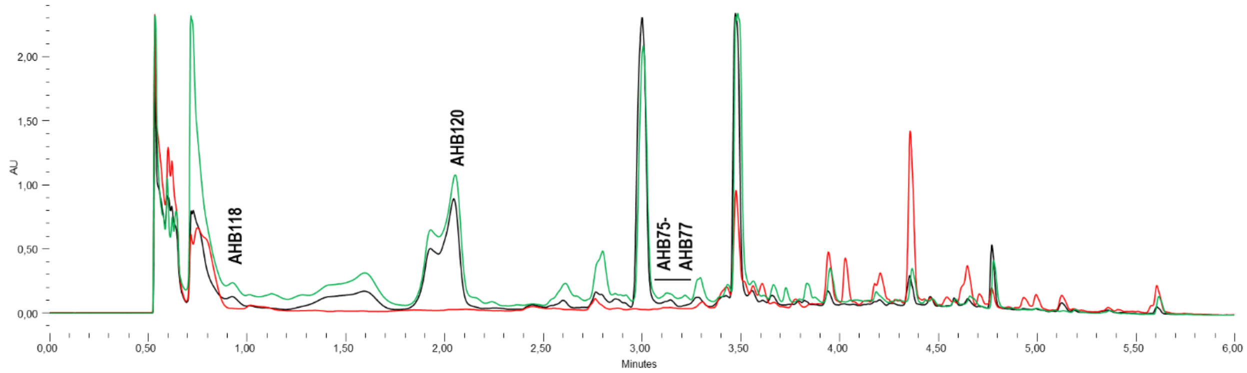

Figure S4. UPLC analysis of extracts of complemented *S. argillaceus* ΔAHBA mutant. Chromatograms at 230 nm of extracts of *S. argillaceus* WT-pREG (black line), *S. argillaceus* ΔAHBA-pREG (red line) and *S. argillaceus* ΔAHBA-pREG-pSETeAHBAHyg (green line).

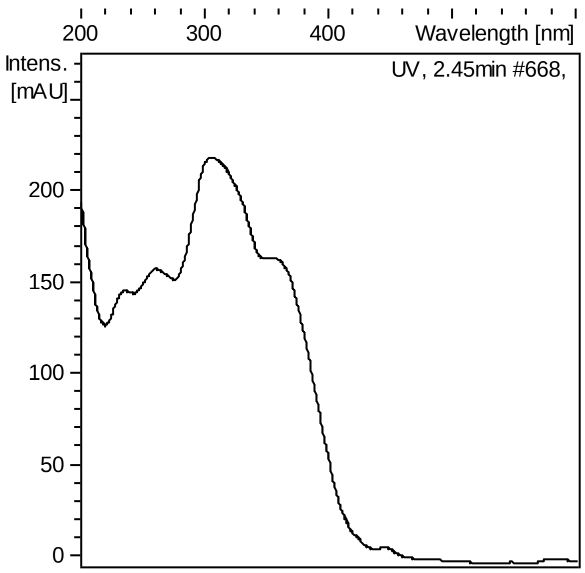

Figure S5. UV-vis (DAD) spectrum of **AHB18**.

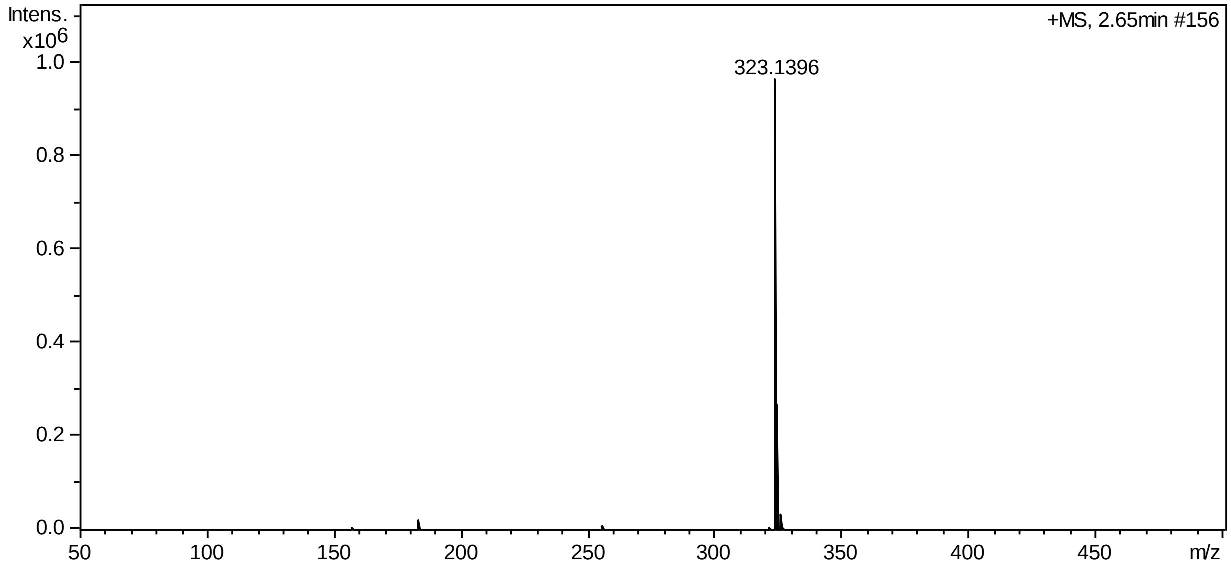

Figure S6. ESI-TOF HRMS spectrum of **AHB18**.

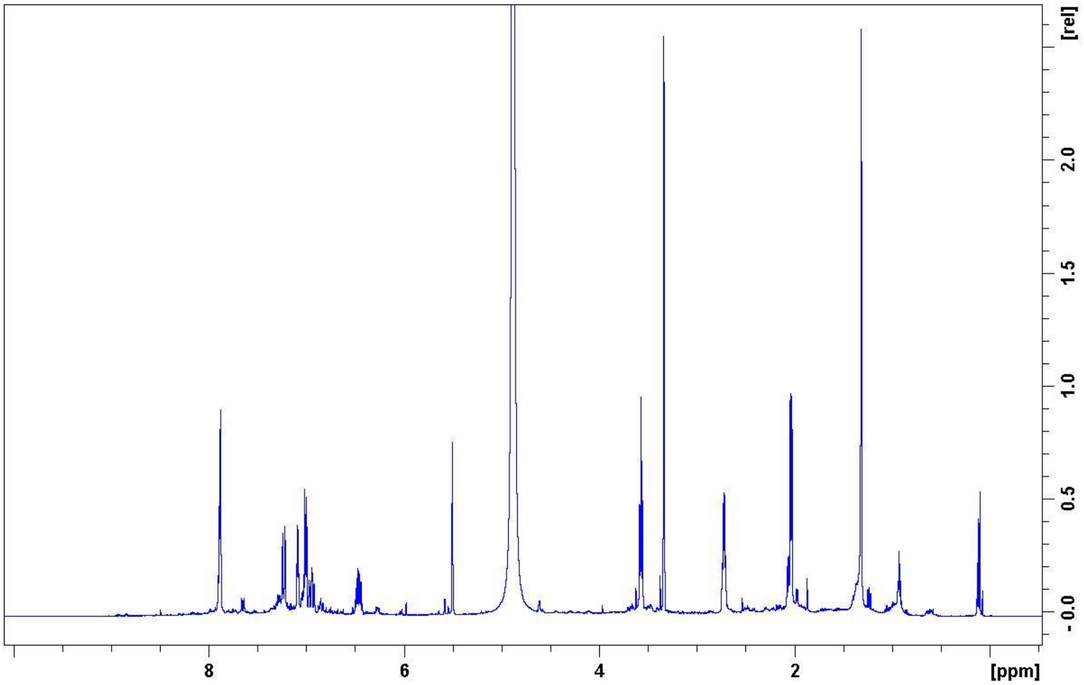

Figure S7. ^{1}H spectrum of **AHB18** (CD_3OD, 24 °C, 500 MHz).

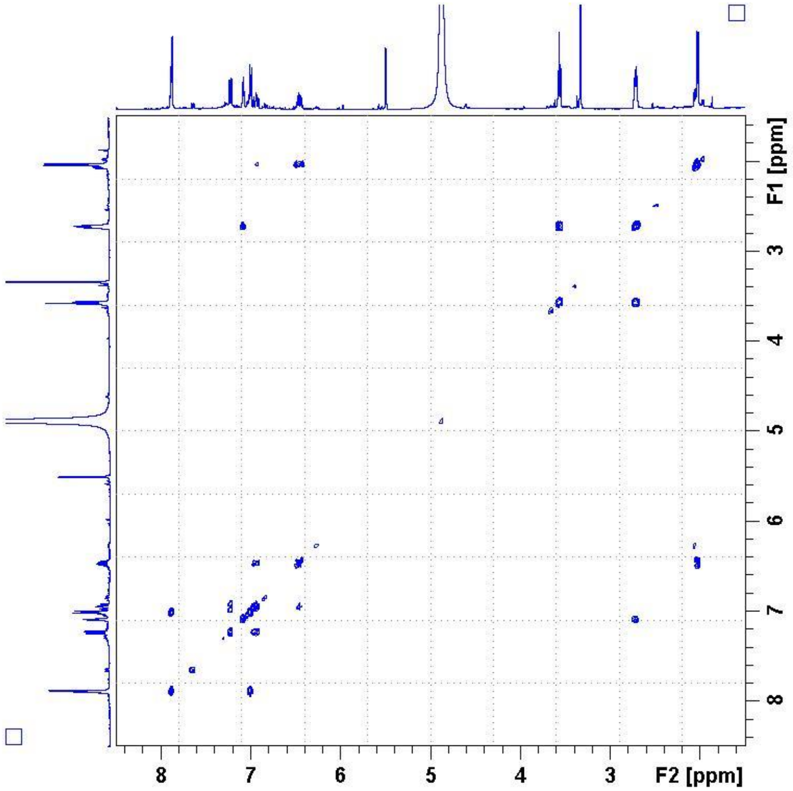

Figure S8. COSY spectrum of **AHB18**.

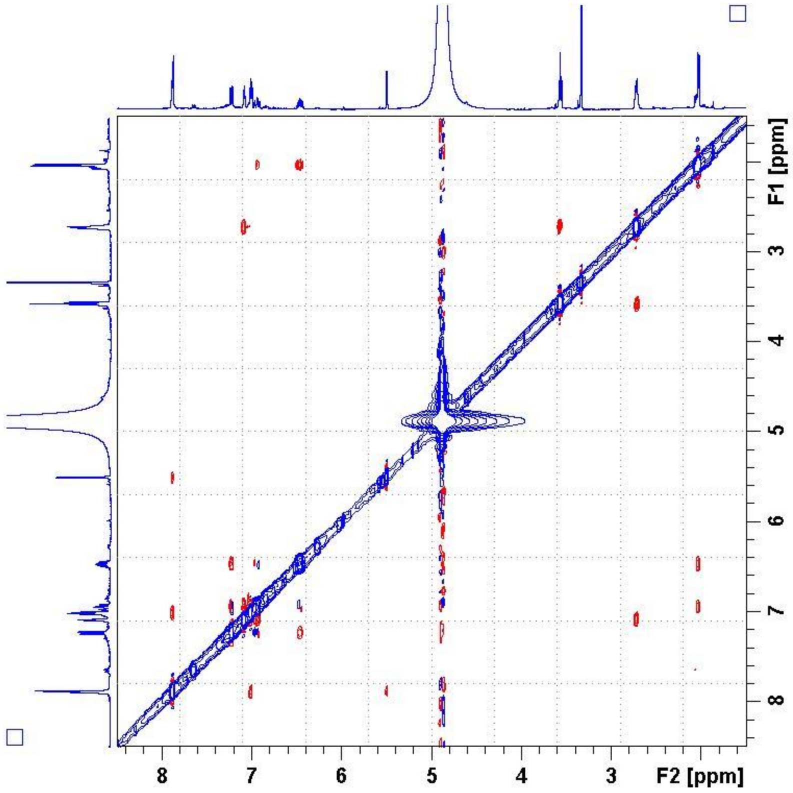

Figure S9. NOESY spectrum of **AHB18**.

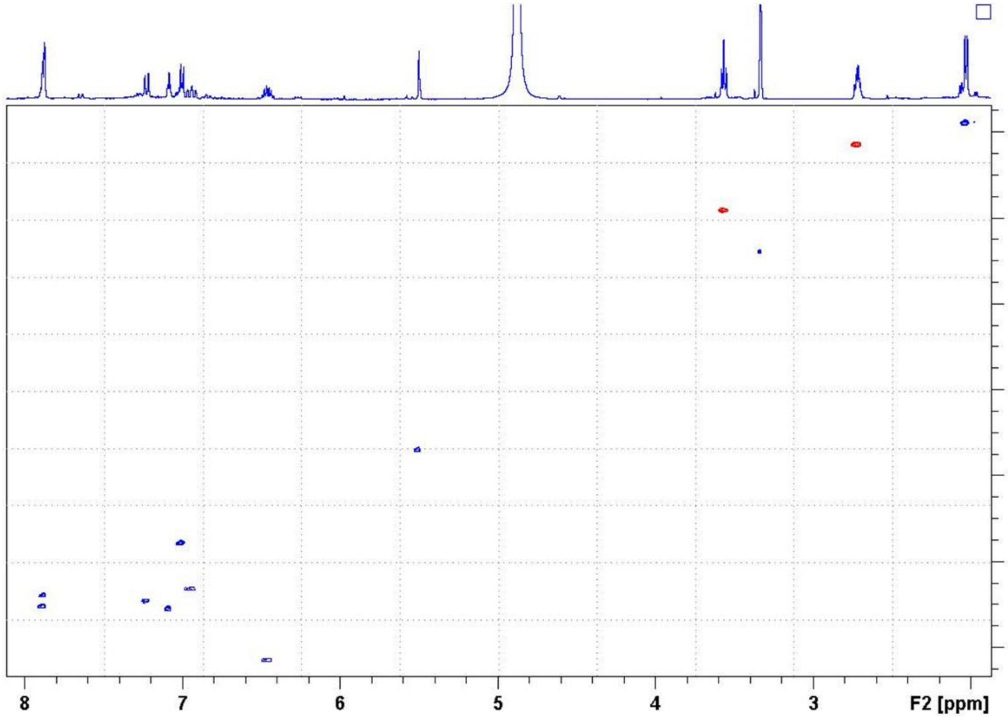

Figure S10. HSQC spectrum of **AHB18**.

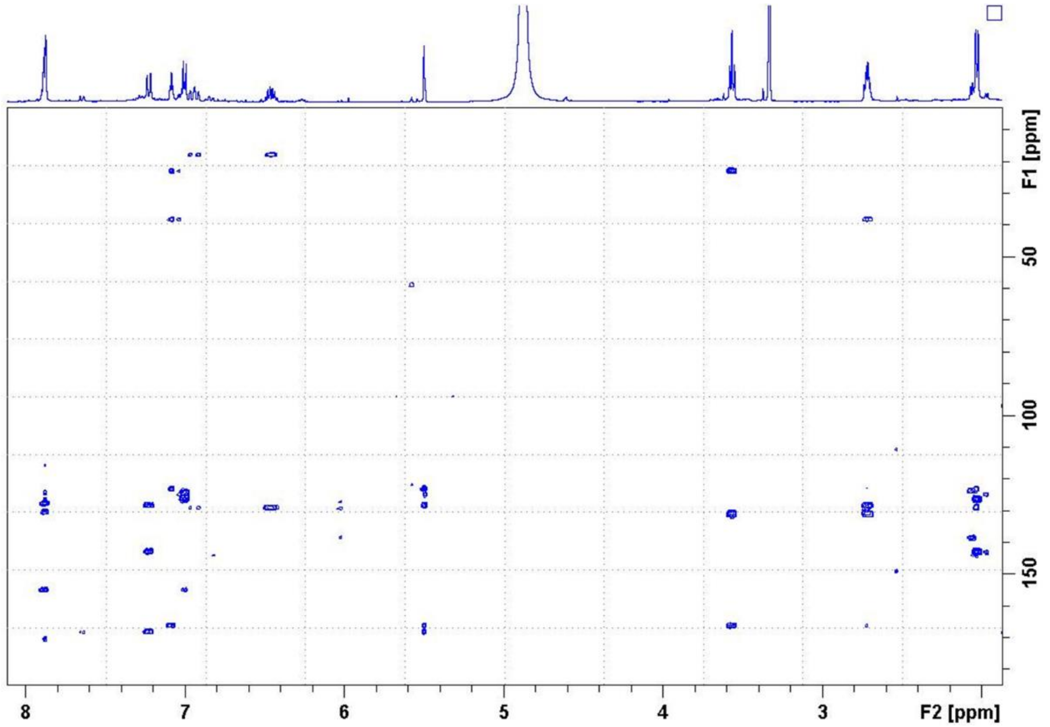

Figure S11. ^{1}H-^{13}C HMBC spectrum of **AHB18**.

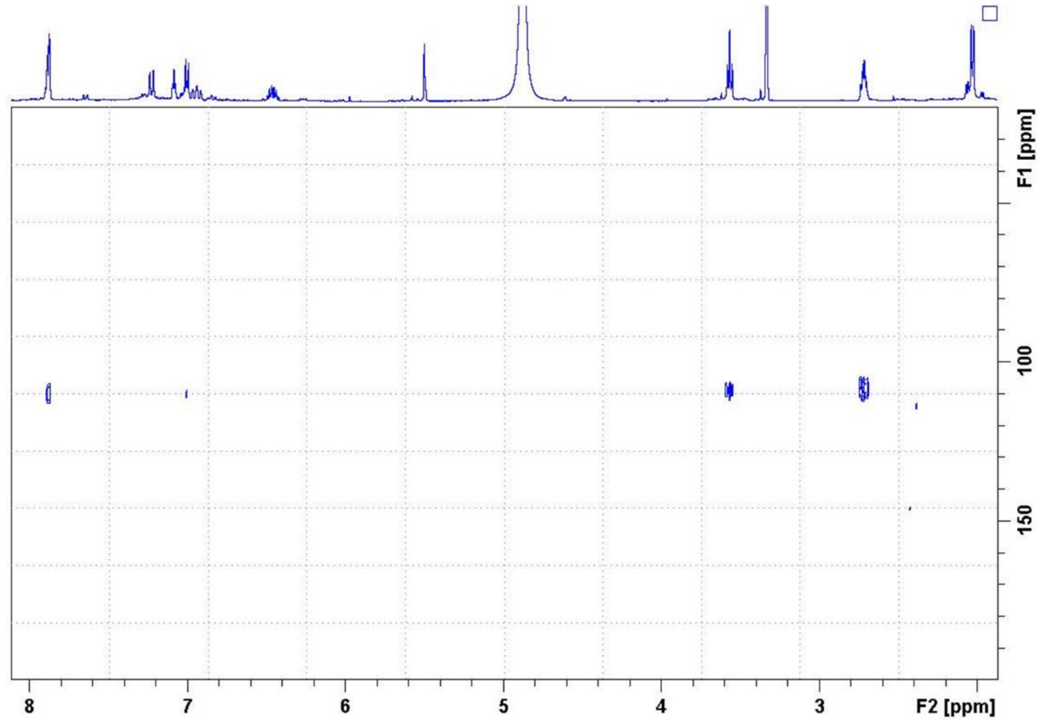

Figure S12. ^{1}H-^{15}N HMBC spectrum of **AHB18**.

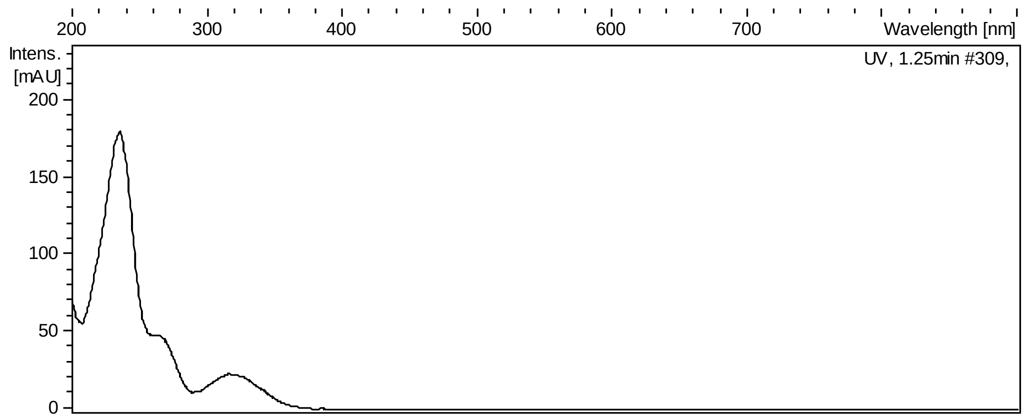

Figure S13. UV-vis (DAD) spectrum of **AHB74**.

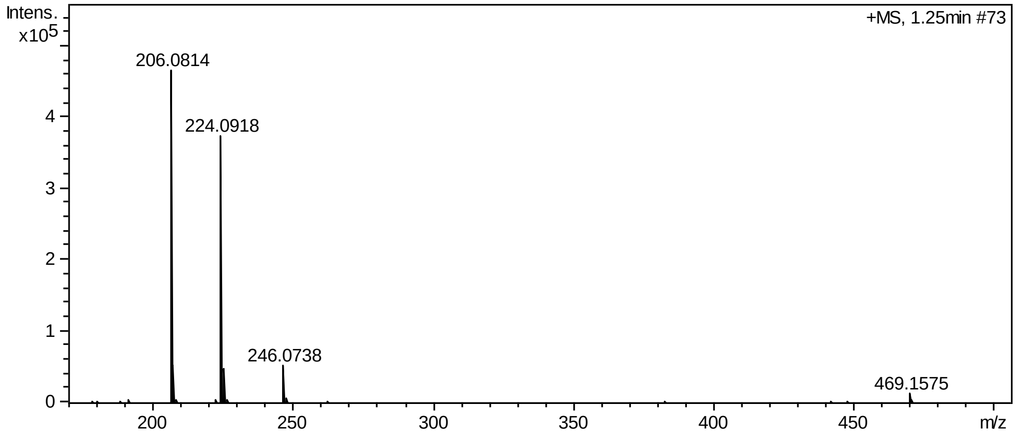

Figure S14. ESI-TOF HRMS spectrum of **AHB74**.

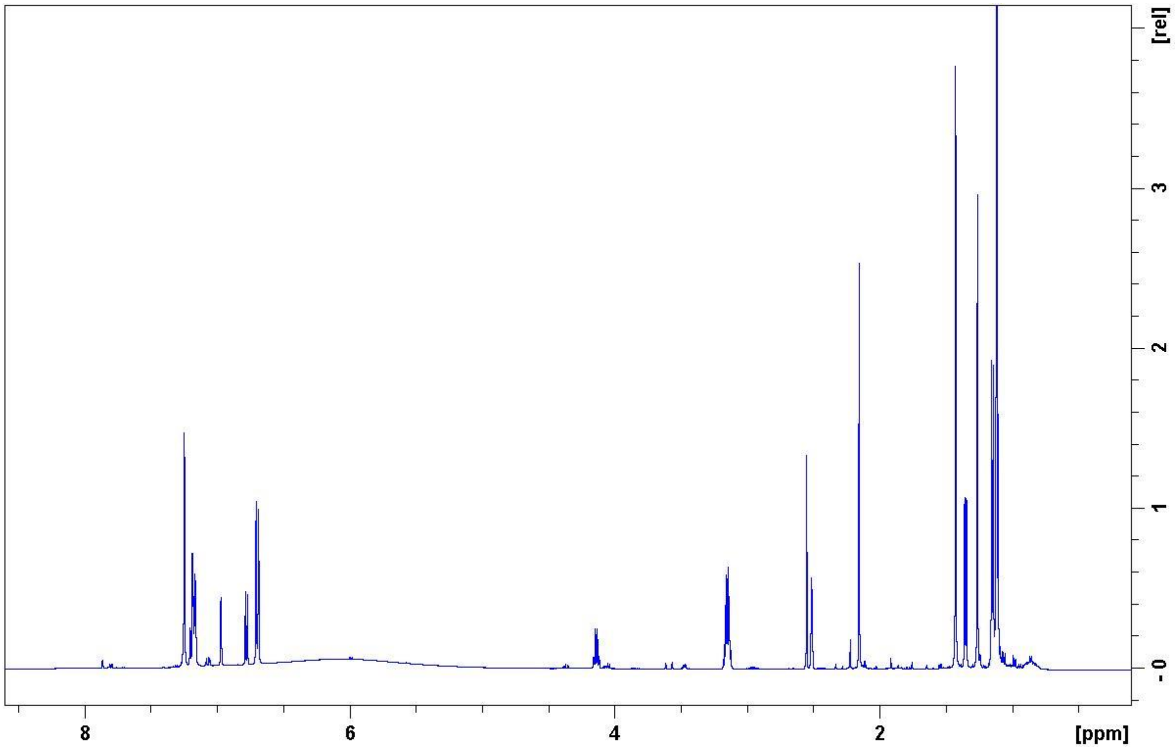

Figure S15. ^{1}H spectrum of **AHB74** (DMSO-d_6, 24 °C, 500 MHz).

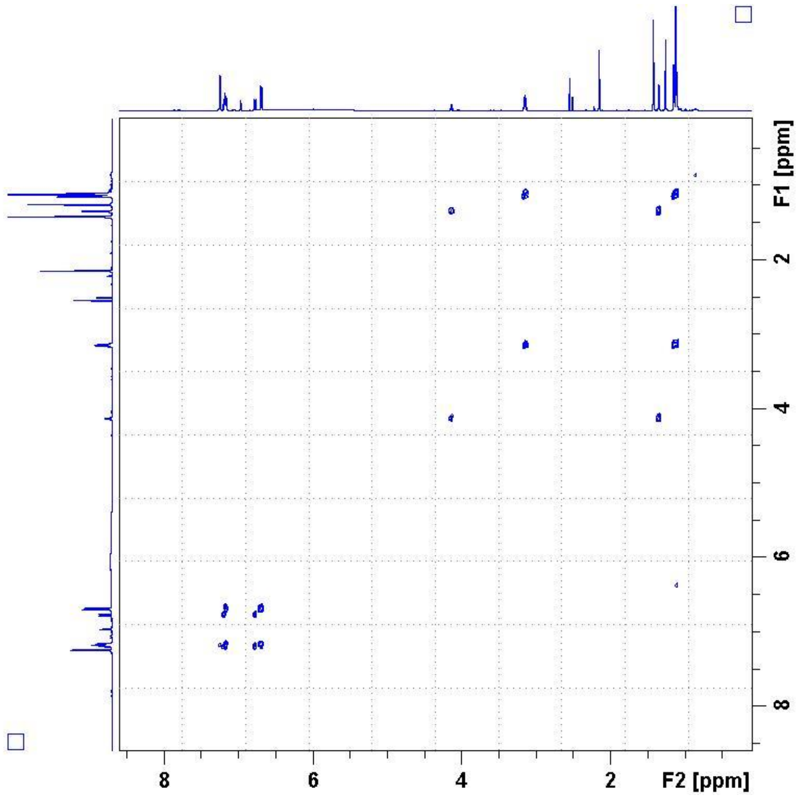

Figure S16. COSY spectrum of **AHB74**.

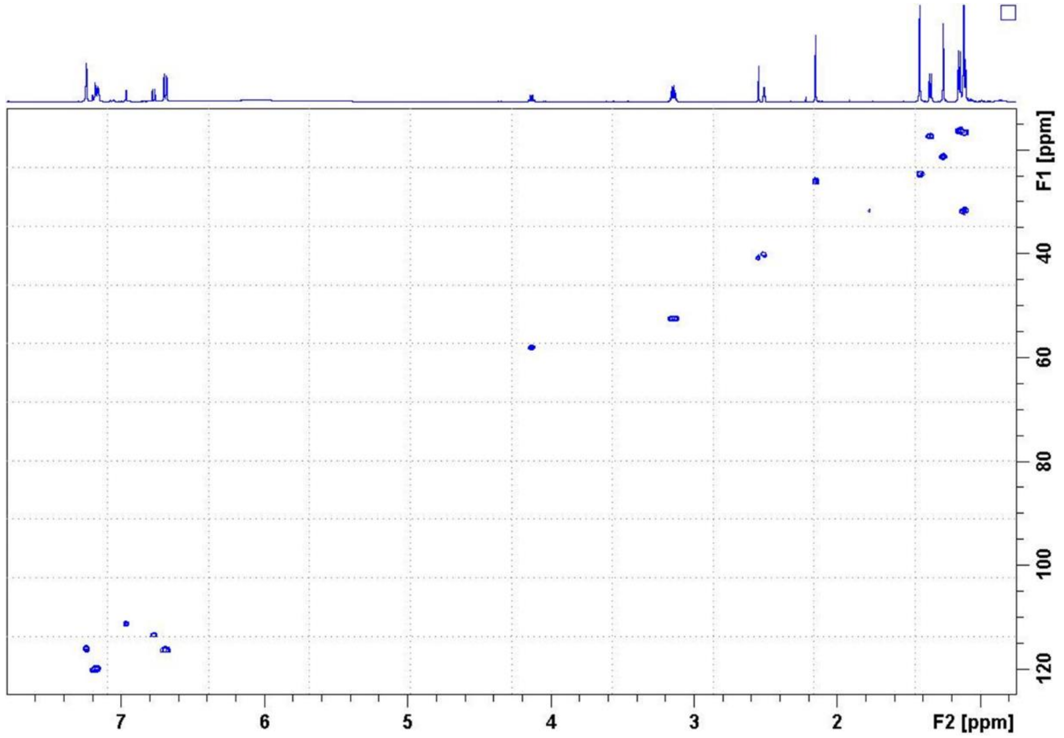

Figure S17. HSQC spectrum of **AHB74**.

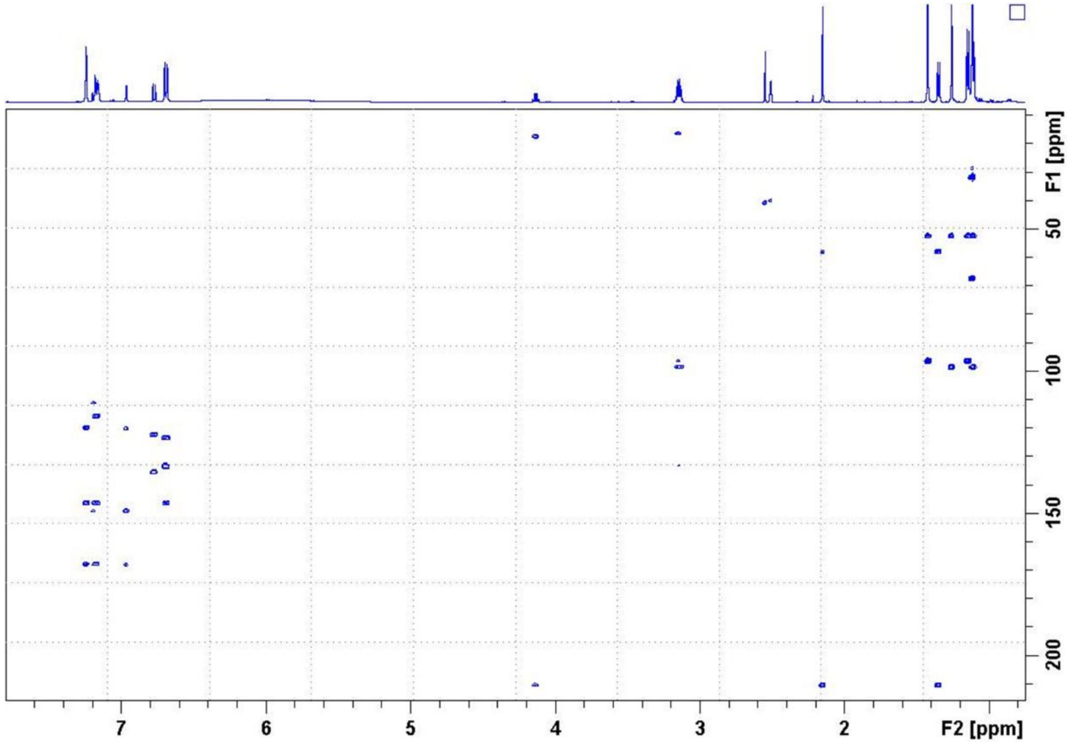

Figure S18. HMBC spectrum of **AHB74**.

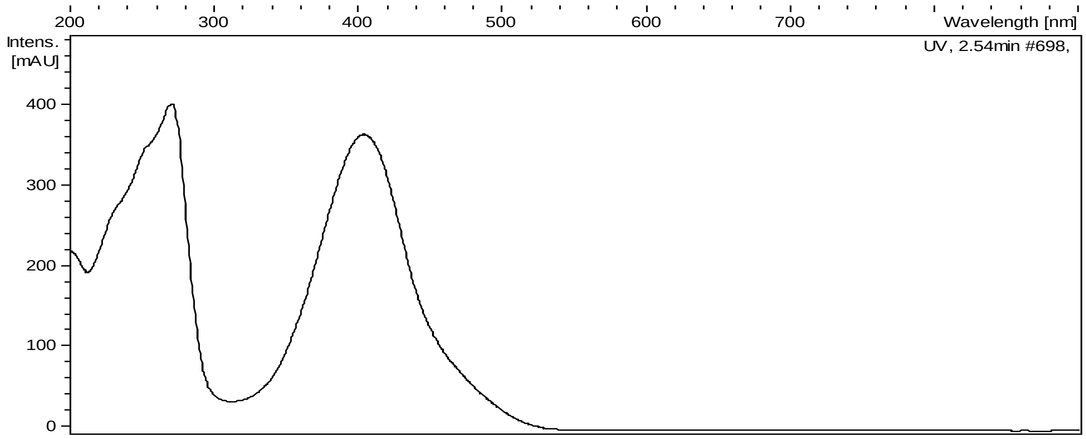

Figure S19. UV-vis (DAD) spectrum of **AHB75**.

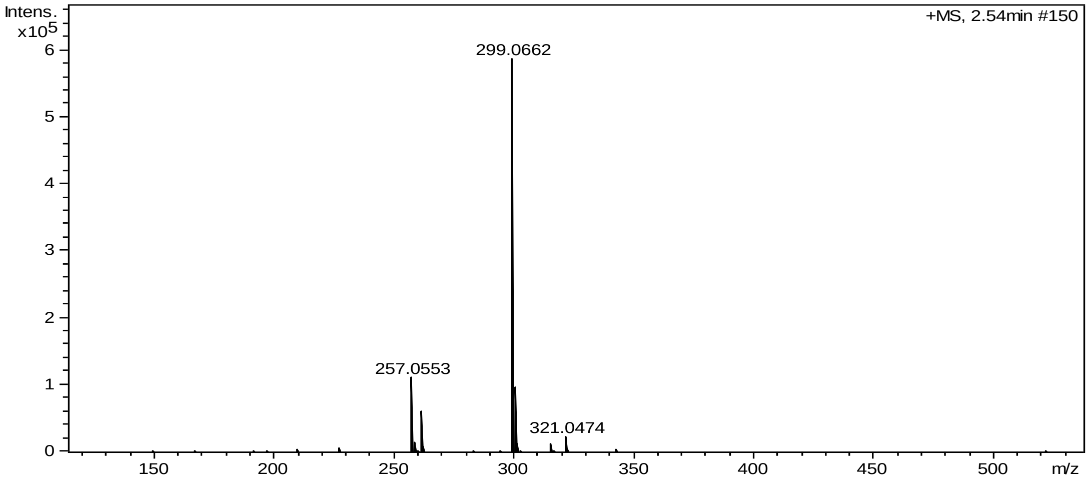

Figure S20. ESI-TOF HRMS spectrum of **AHB75**.

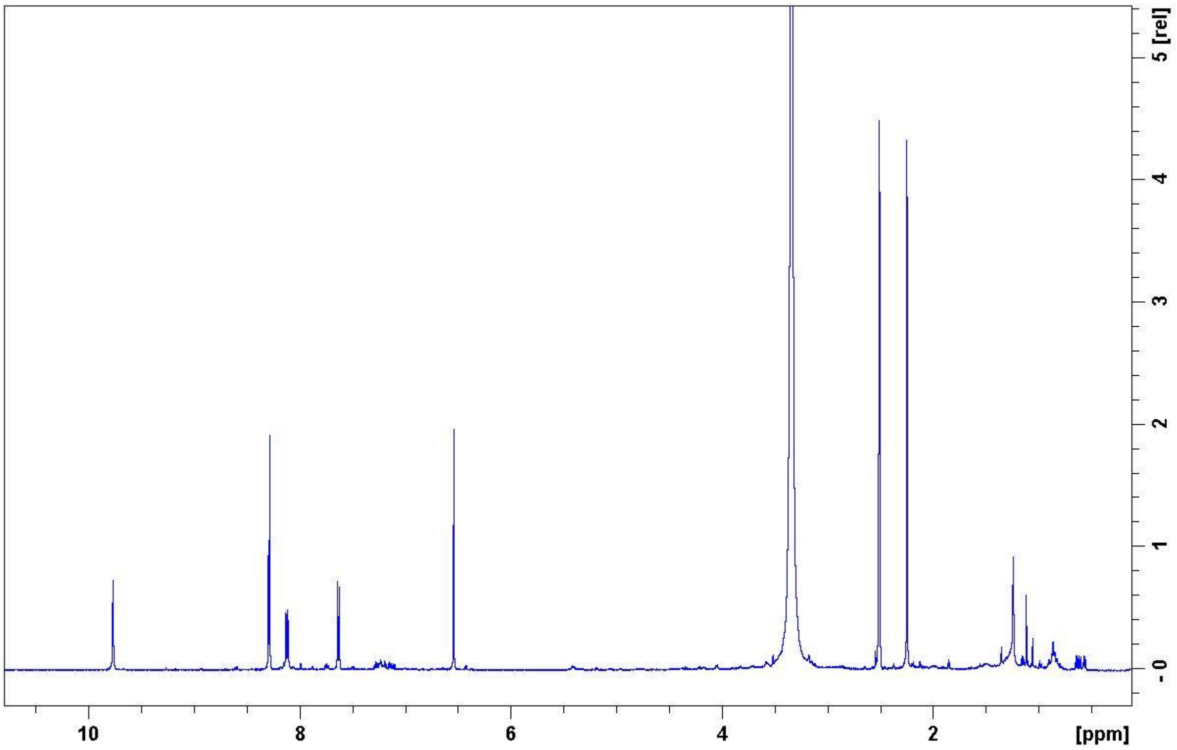

Figure S21. ^{1}H spectrum of **AHB75** (DMSO-d_6, 24 °C, 500 MHz).

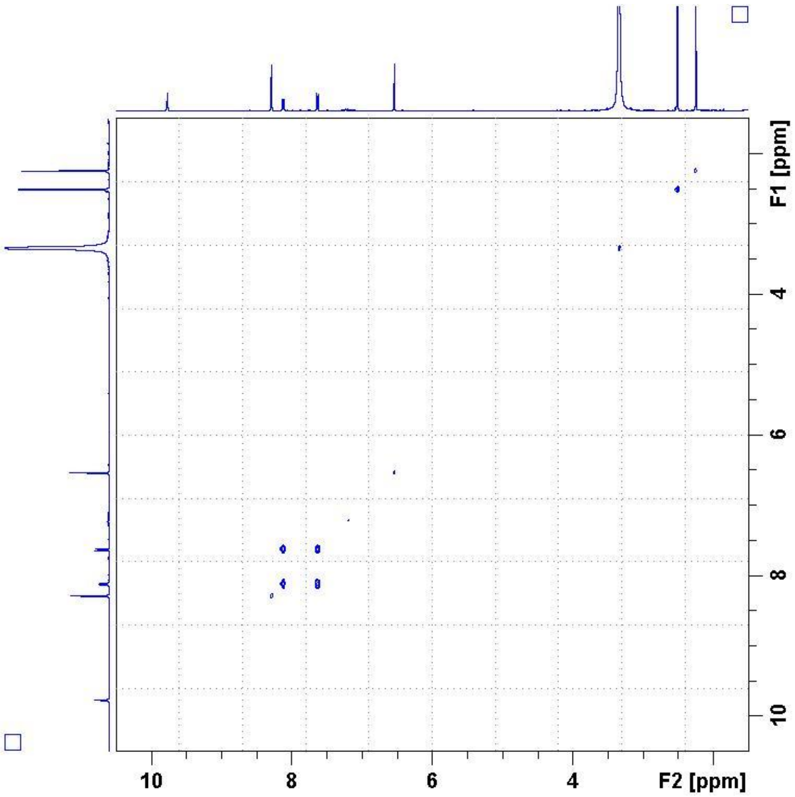

Figure S22. COSY spectrum of **AHB75**.

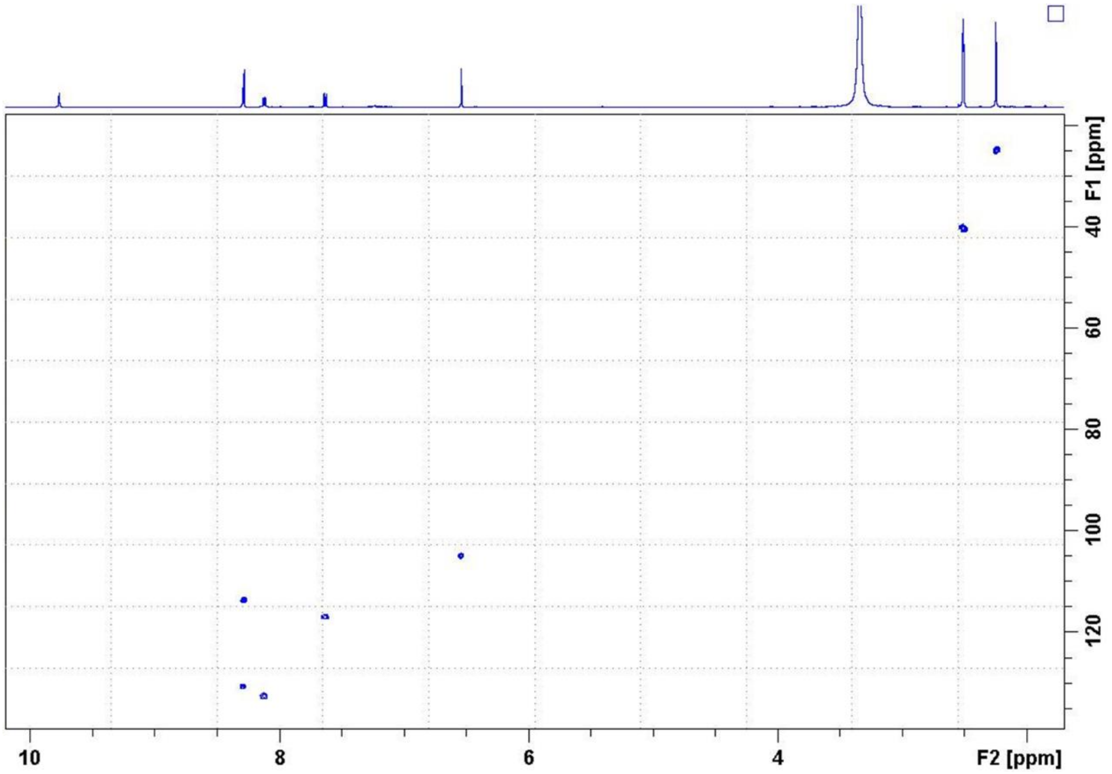

Figure S23. HSQC spectrum of **AHB75**.

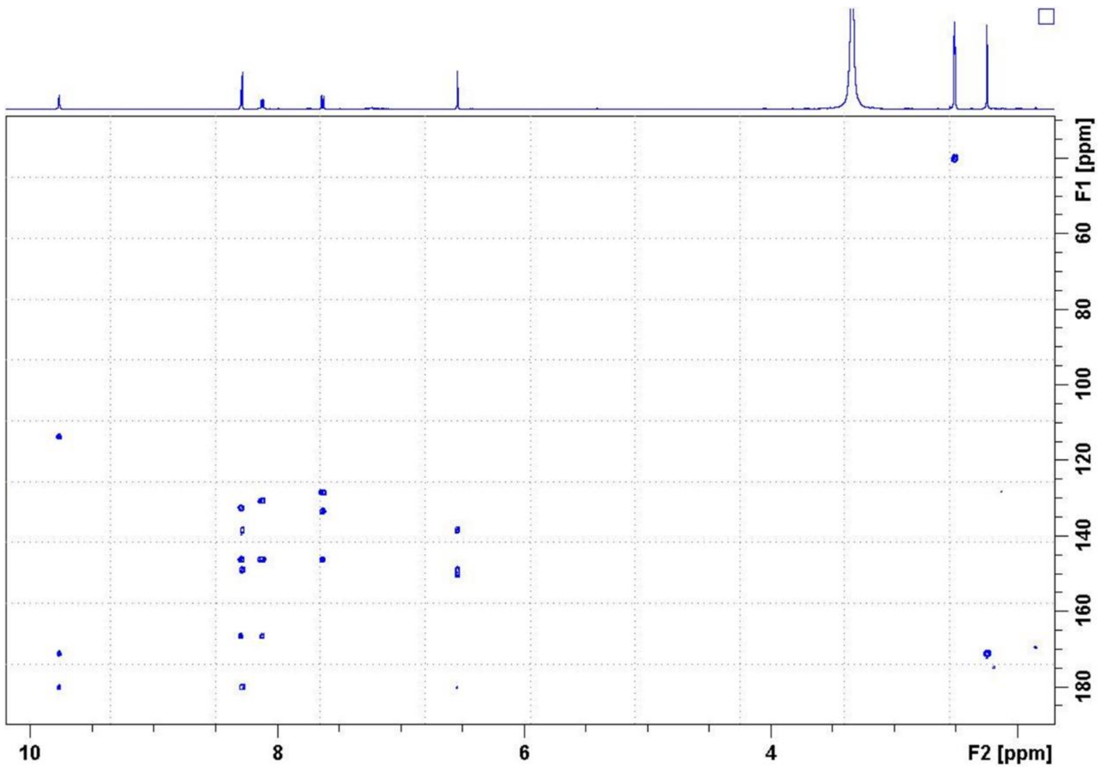

Figure S24. HMBC spectrum of **AHB75**.

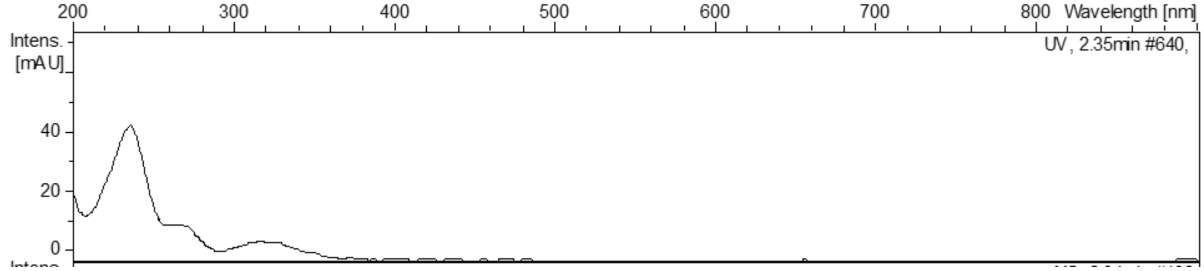

Figure S25. UV-vis (DAD) spectrum of **AHB76**.

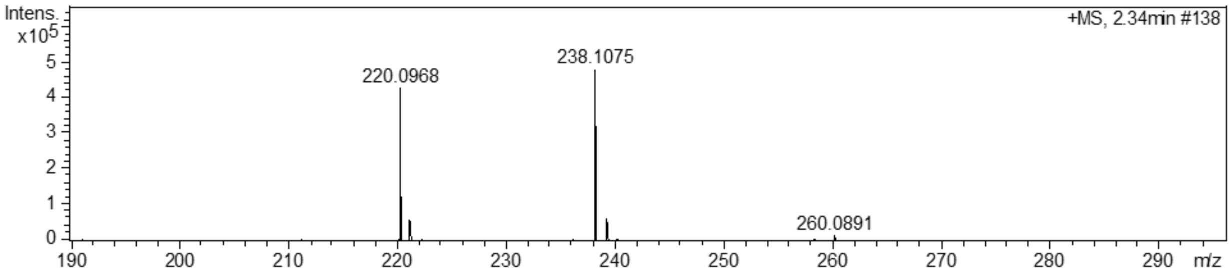

Figure S26. ESI-TOF HRMS spectrum of **AHB76**.

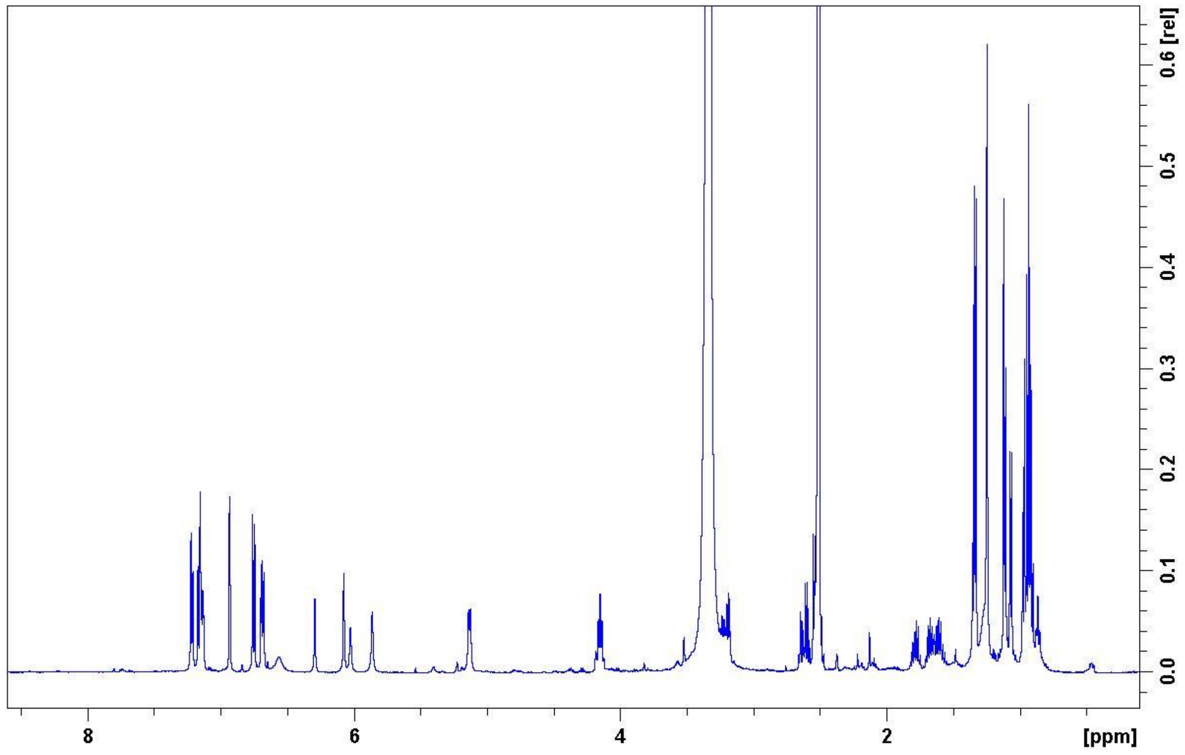

Figure 7 ^{1}H spectrum of **AHB76** (DMSO-d_6, 24 °C, 500 MHz).

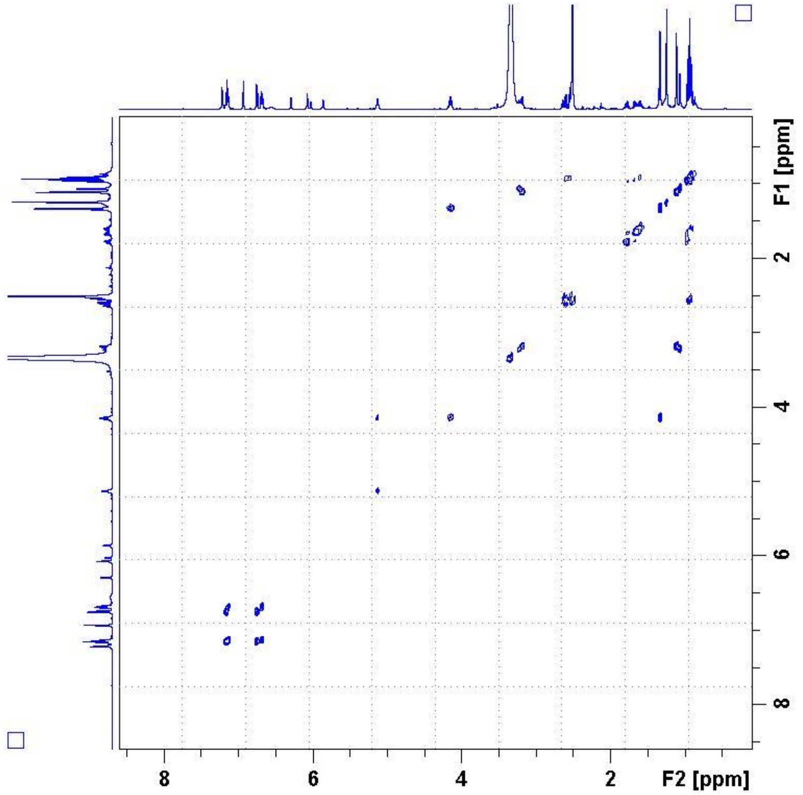

Figure S28. COSY spectrum of **AHB76**.

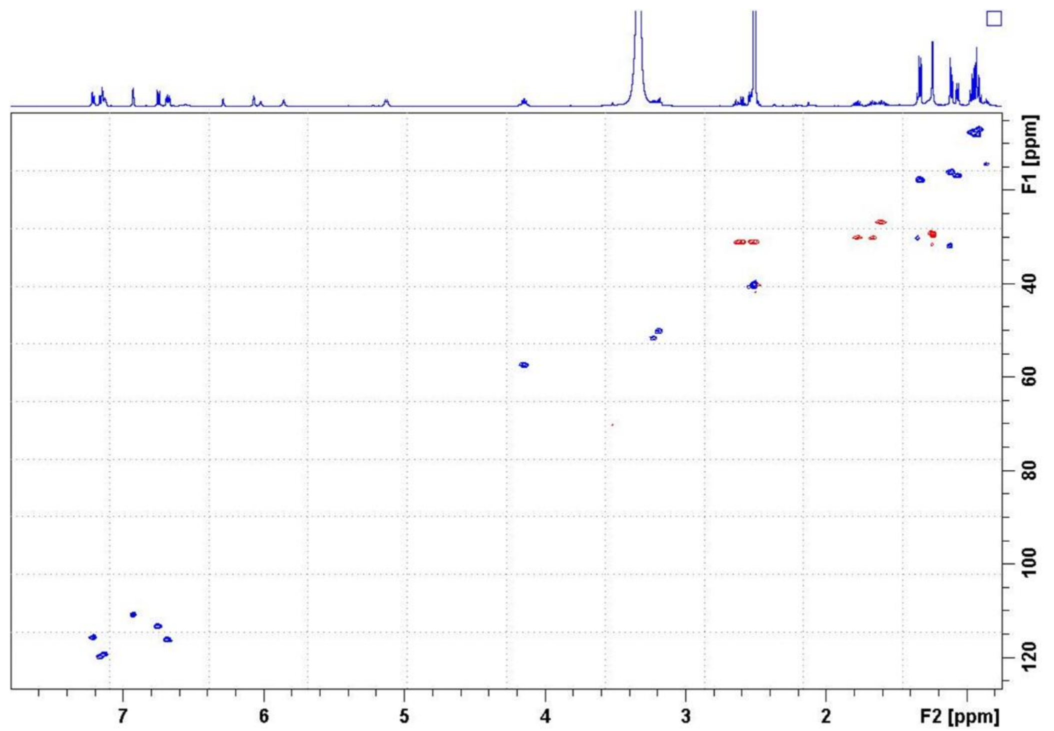

Figure S29. HSQC spectrum of **AHB76**.

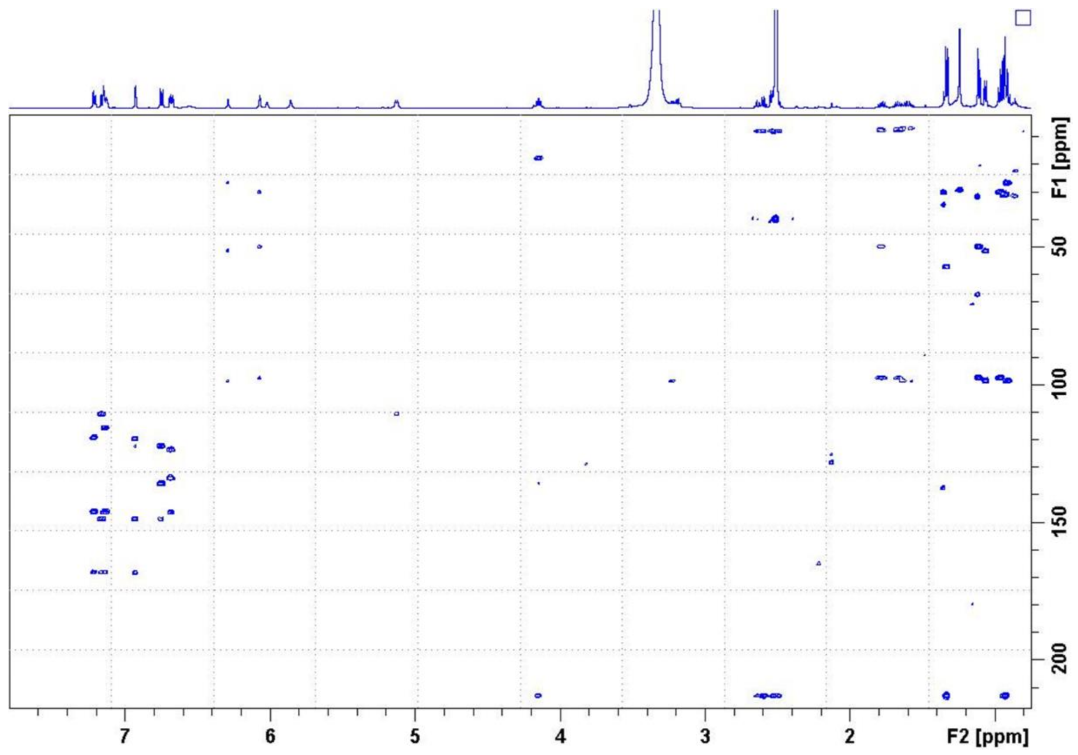

Figure S309. HMBC spectrum of **AHB76**.

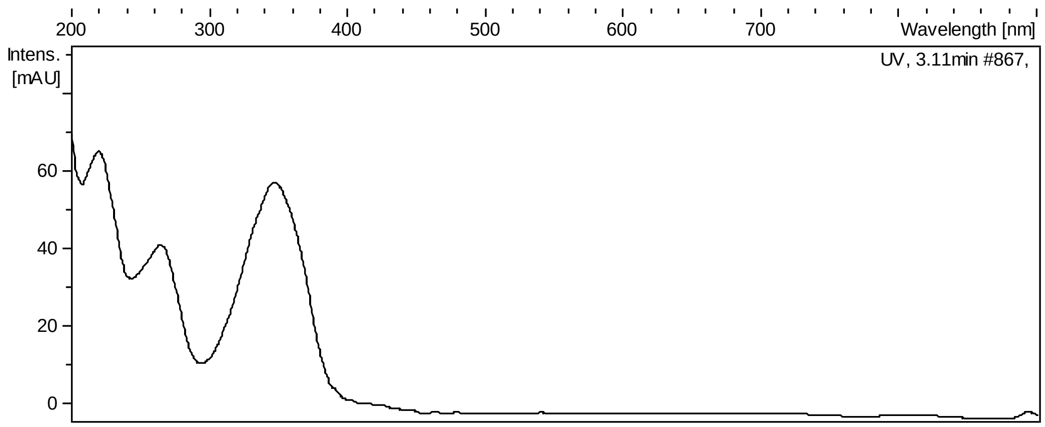

Figure S31. UV-vis (DAD) spectrum of **AHB77**.

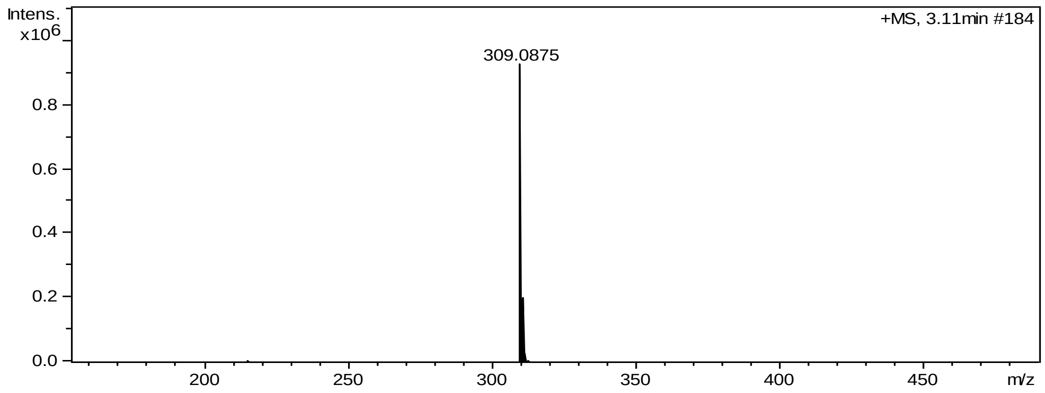

Figure S32. ESI-TOF HRMS spectrum of **AHB77**.

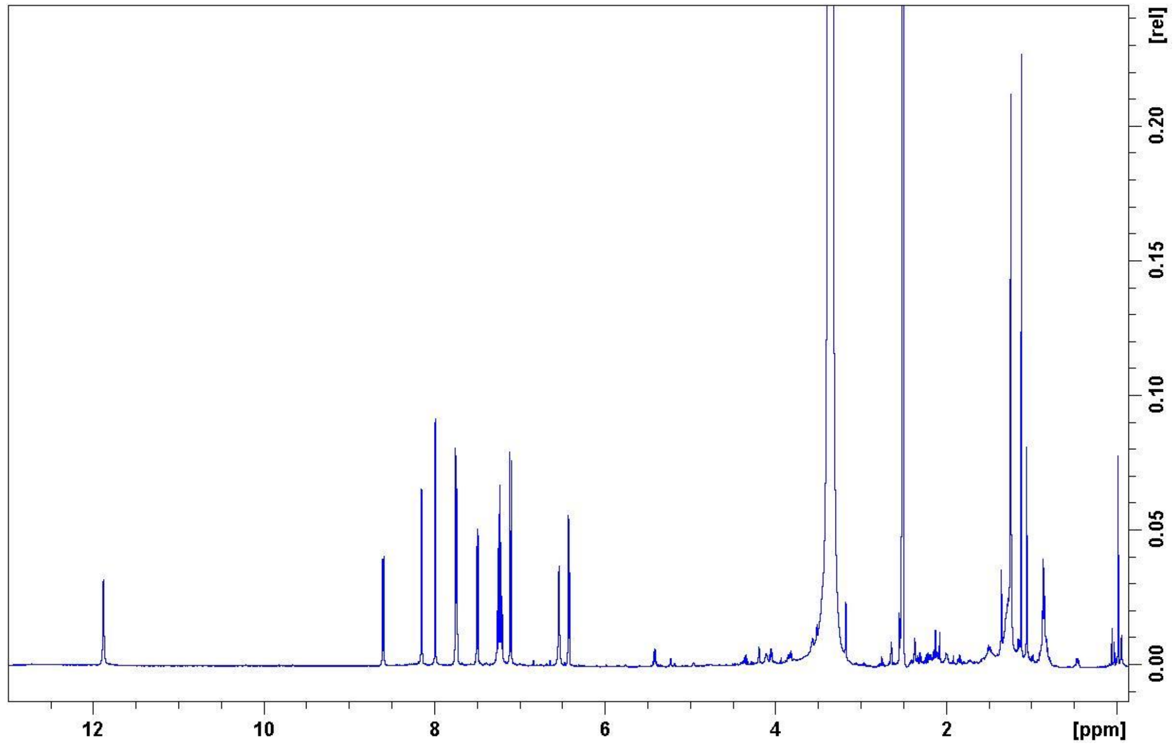

Figure S33. ^{1}H spectrum of **AHB77** (DMSO-d$_6$, 24 °C, 500 MHz).

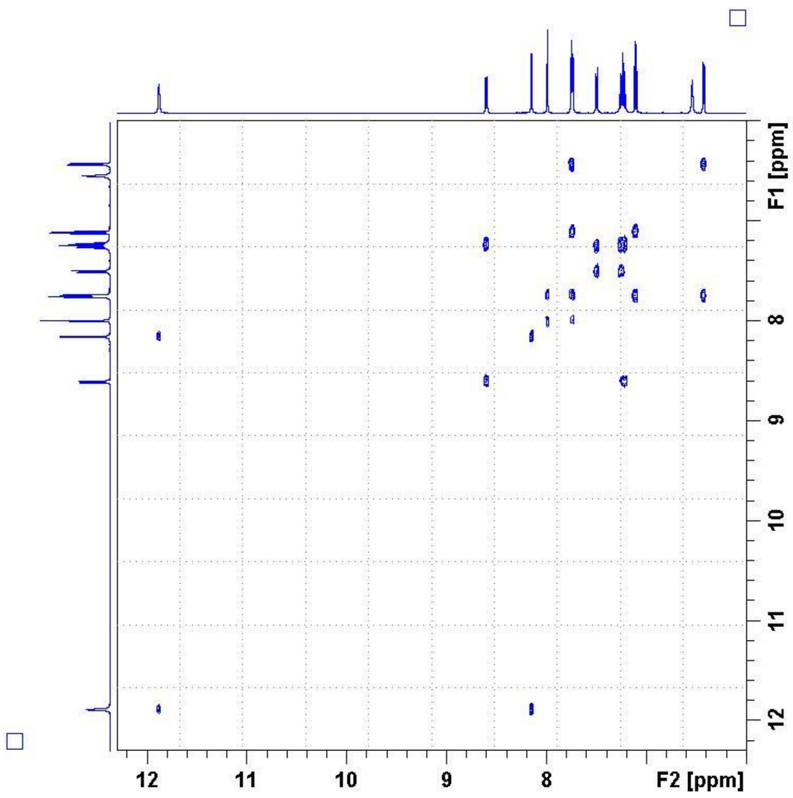

Figure S34. COSY spectrum of **AHB77**.

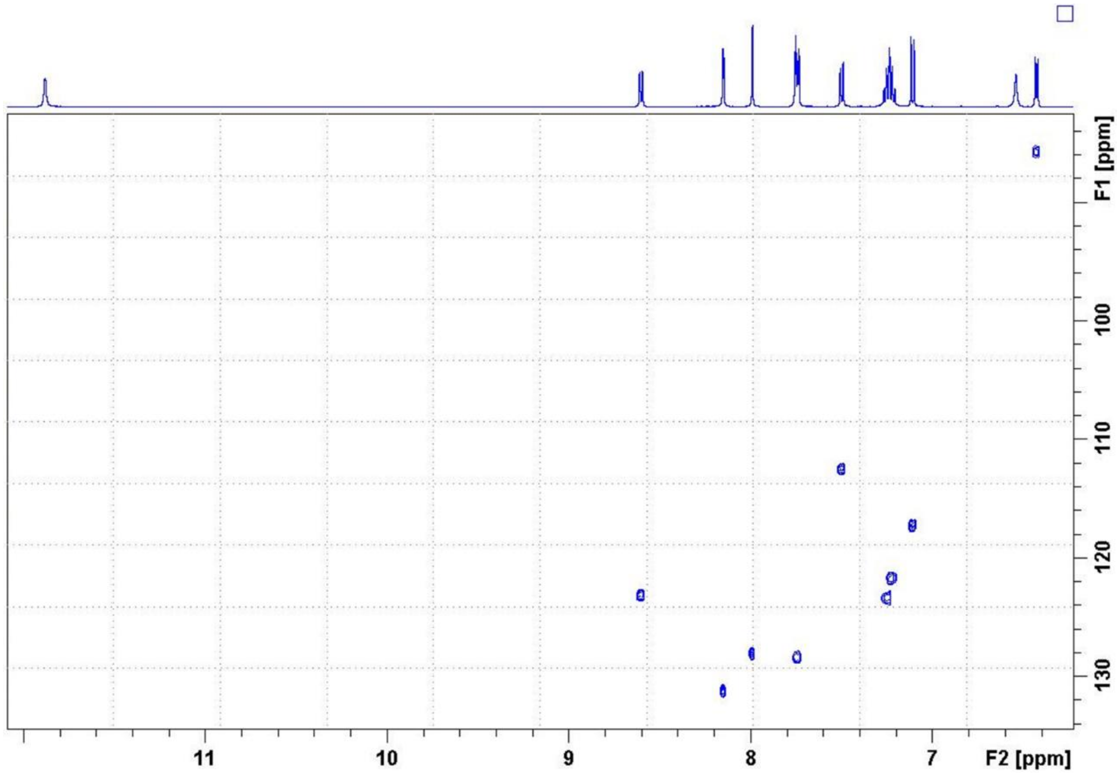

Figure S35. HSQC spectrum of **AHB77**.

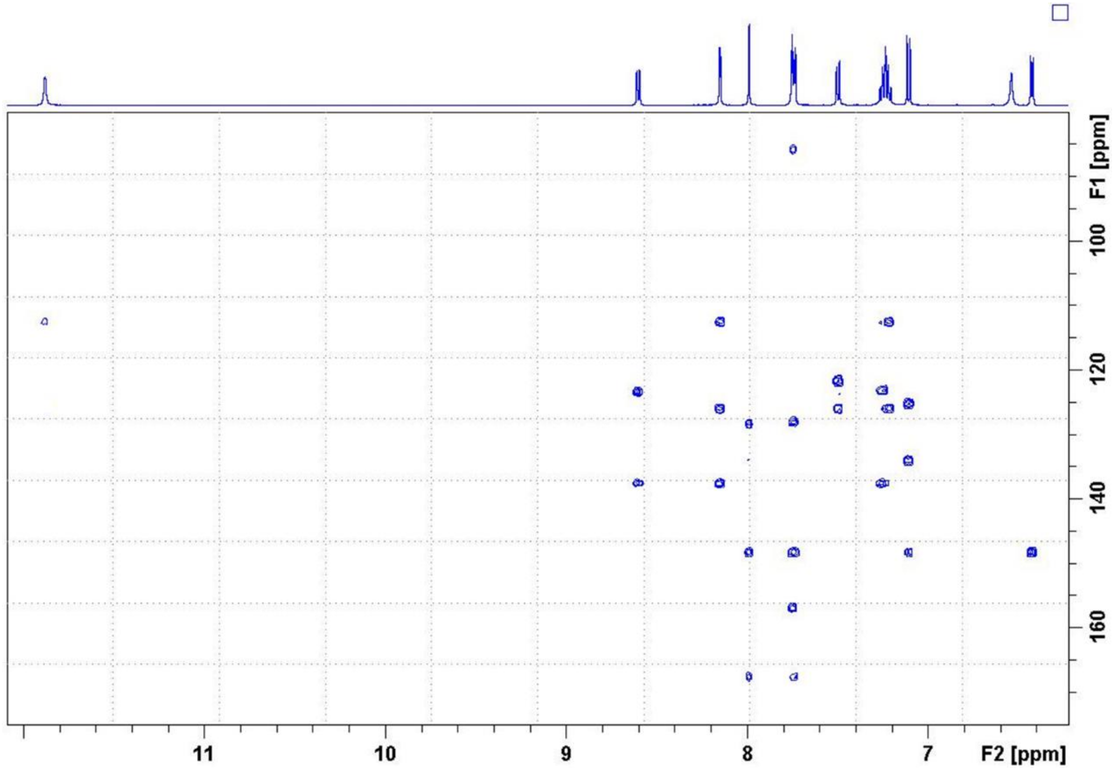

Figure S36. HMBC spectrum of **AHB77**.

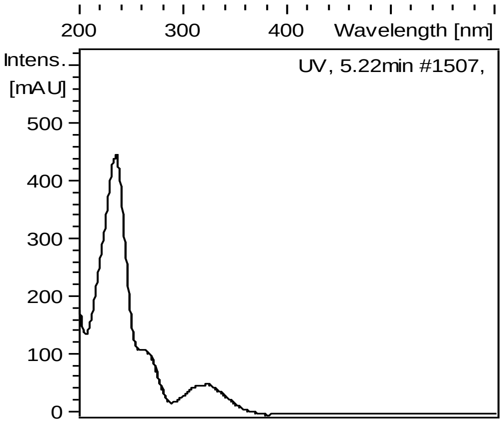

Figure S37. UV-vis (DAD) spectrum of **AHB118**.

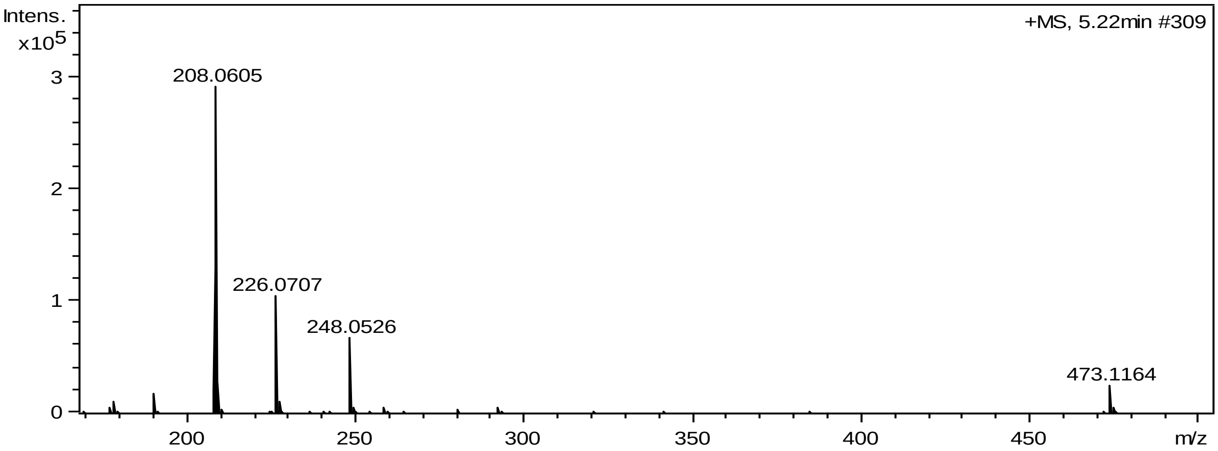

Figure S38. ESI-TOF HRMS spectrum of **AHB118**.

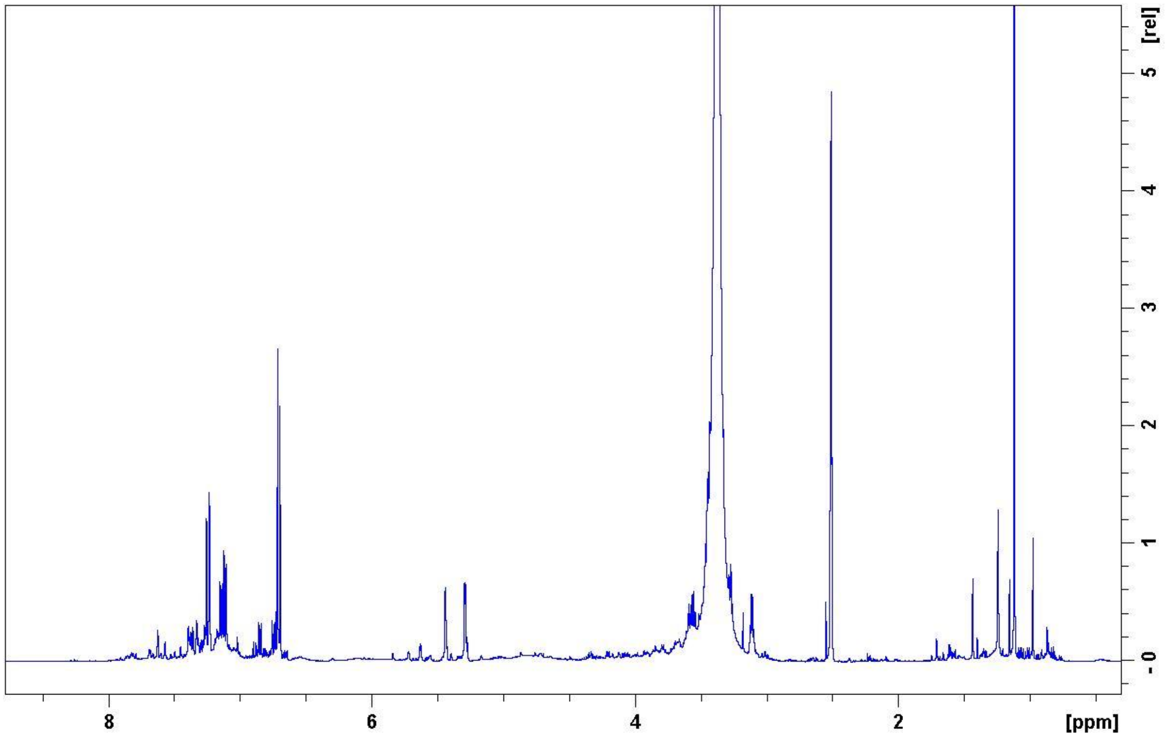

Figure S39. ^{1}H spectrum of **AHB118** ($DMSO-d_6$, 24 °C, 500 MHz).

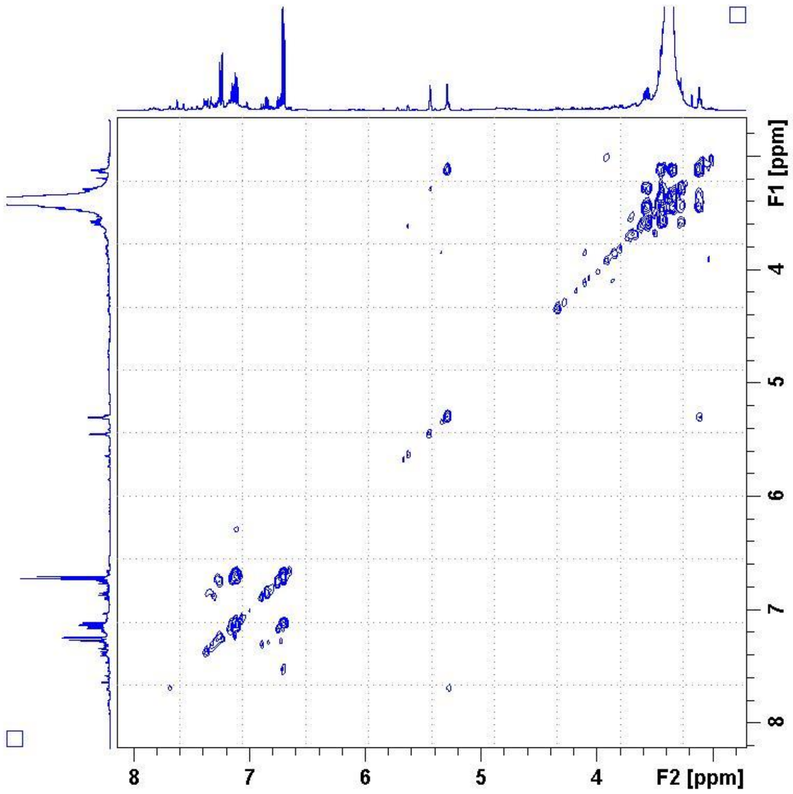

Figure S40. COSY spectrum of **AHB118**.

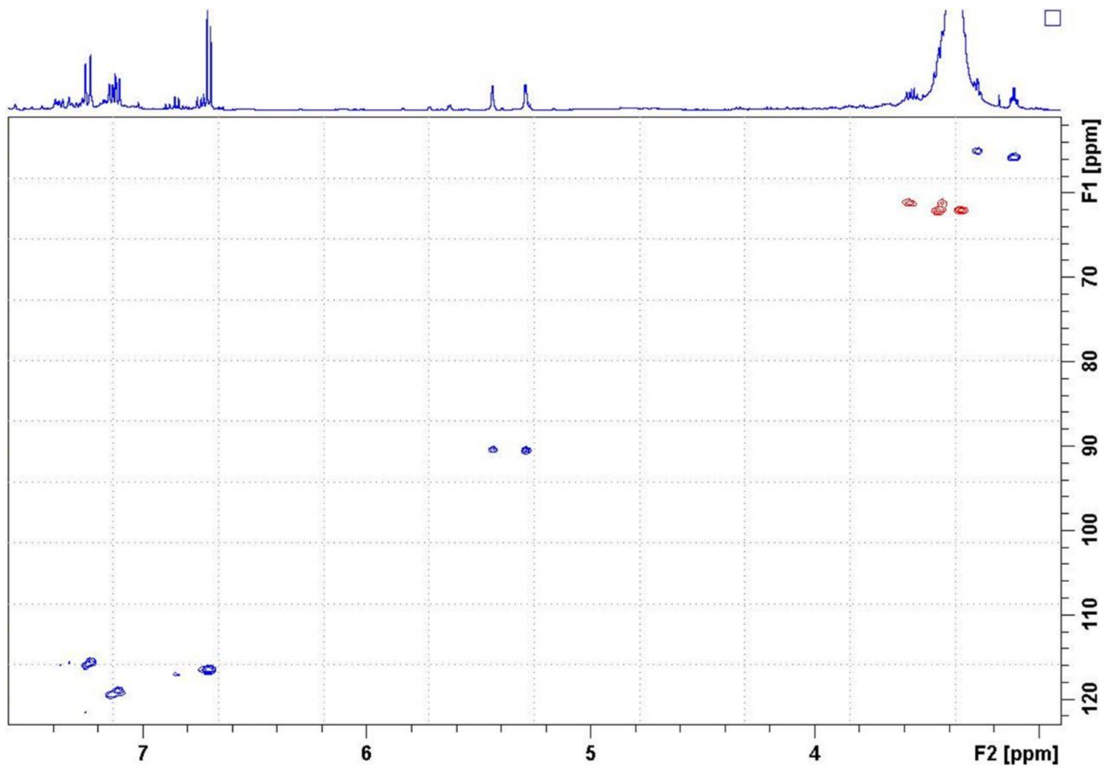

Figure S41. HSQC spectrum of **AHB118**.

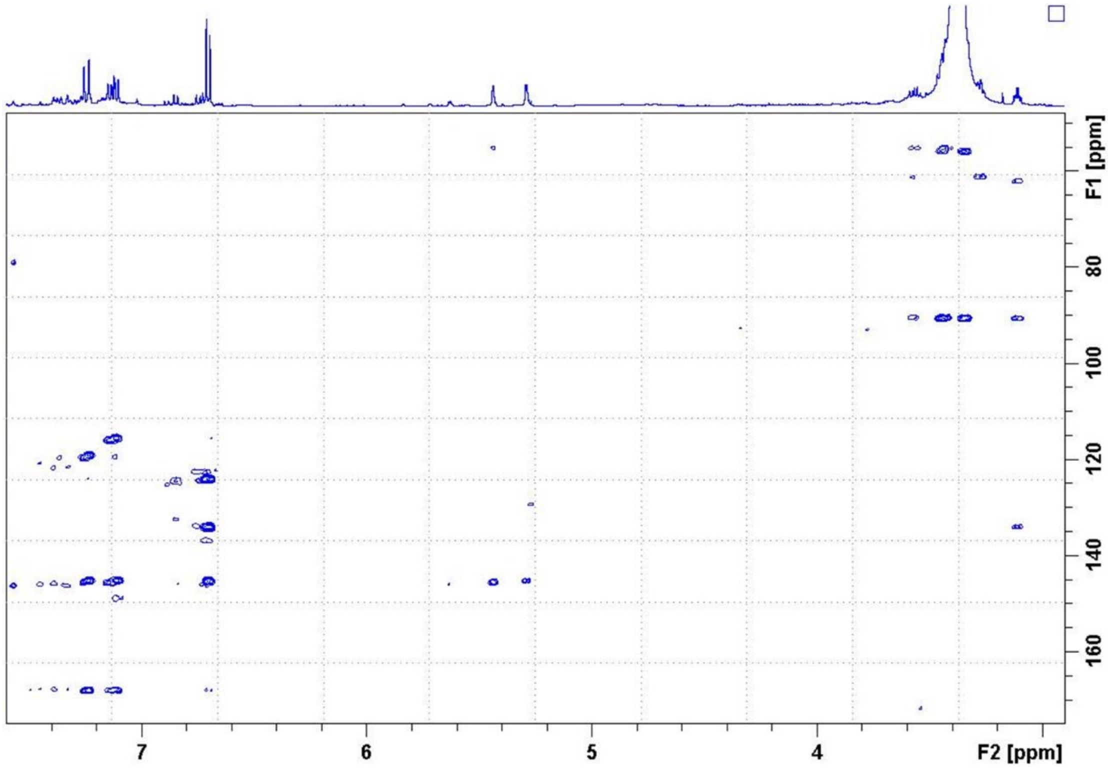

Figure S42. HMBC spectrum of **AHB118**.

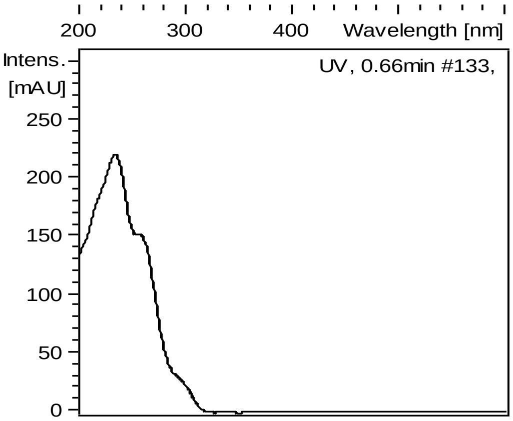

Figure S43. UV-vis (DAD) spectrum of **AHB119**.

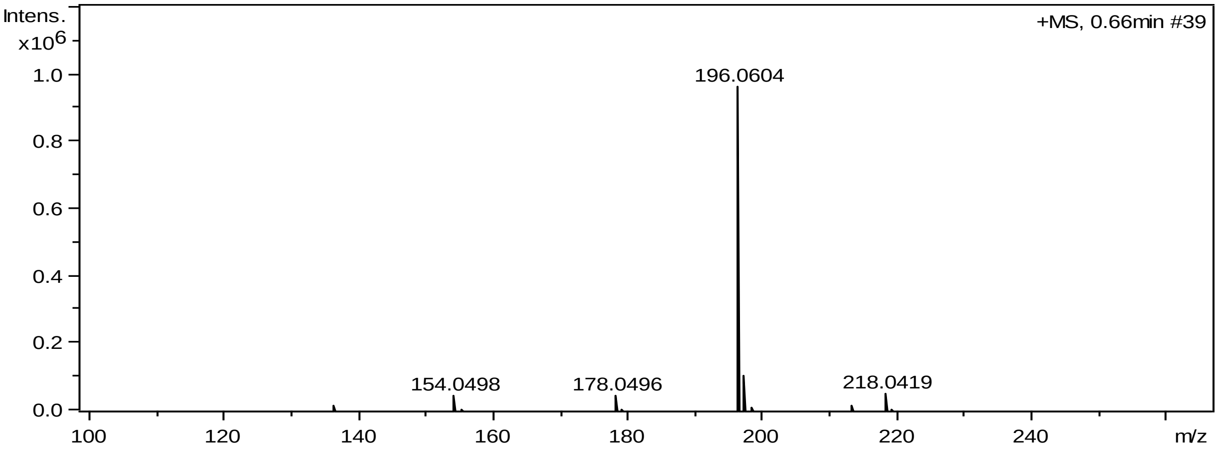

Figure S44. ESI-TOF HRMS spectrum of **AHB119**.

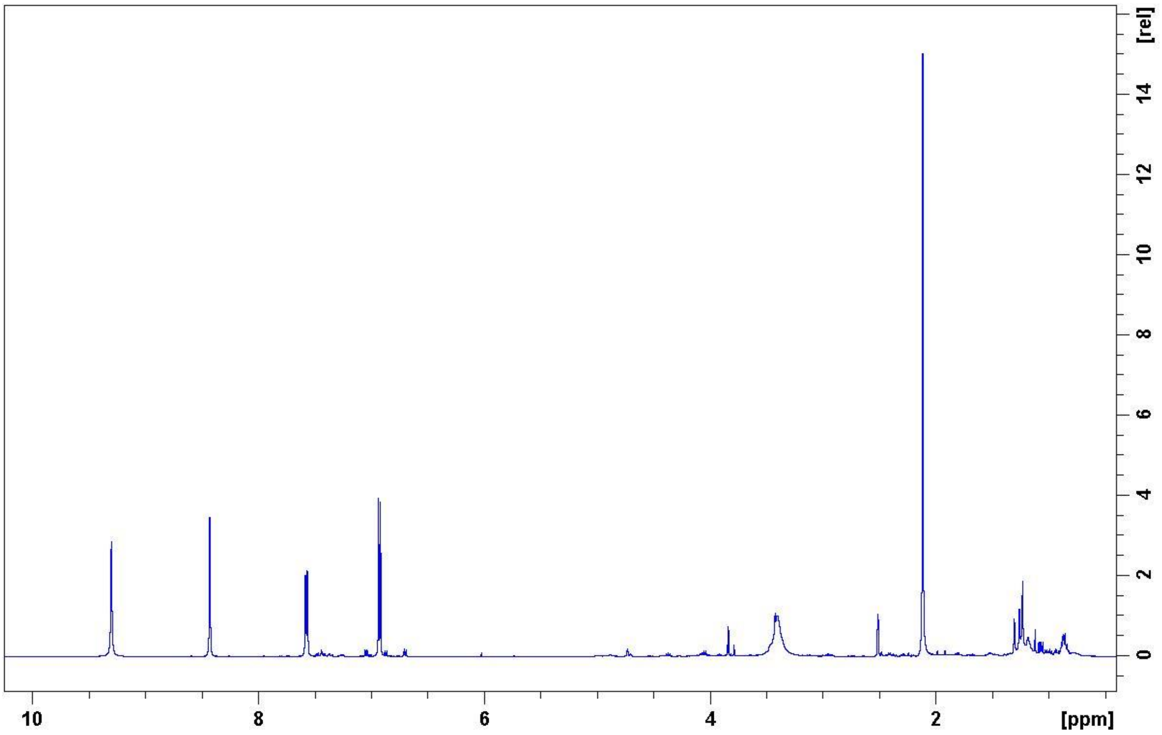

Figure S45. ^{1}H spectrum of **AHB119** (DMSO-d_6, 24 °C, 500 MHz).

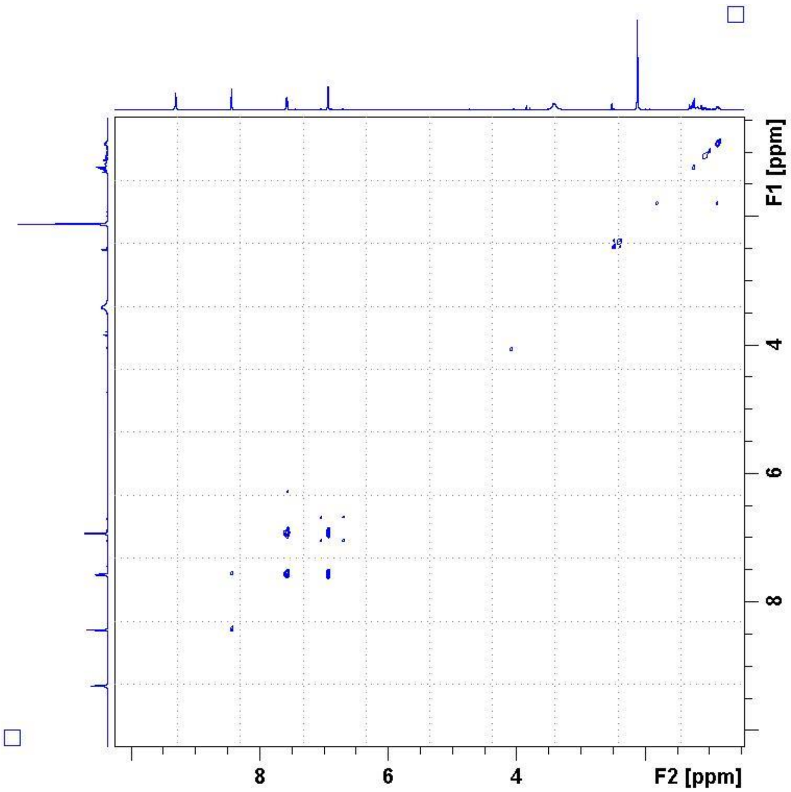

Figure S46. COSY spectrum of **AHB119**.

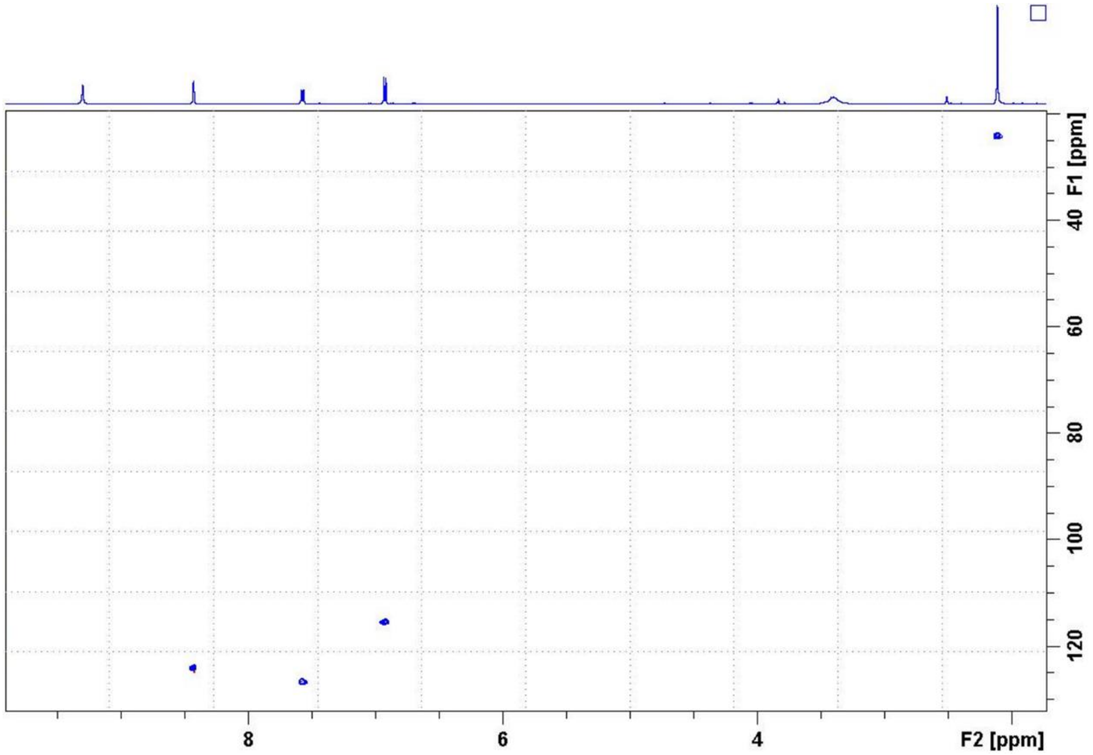

Figure S47. HSQC spectrum of **AHB119**.

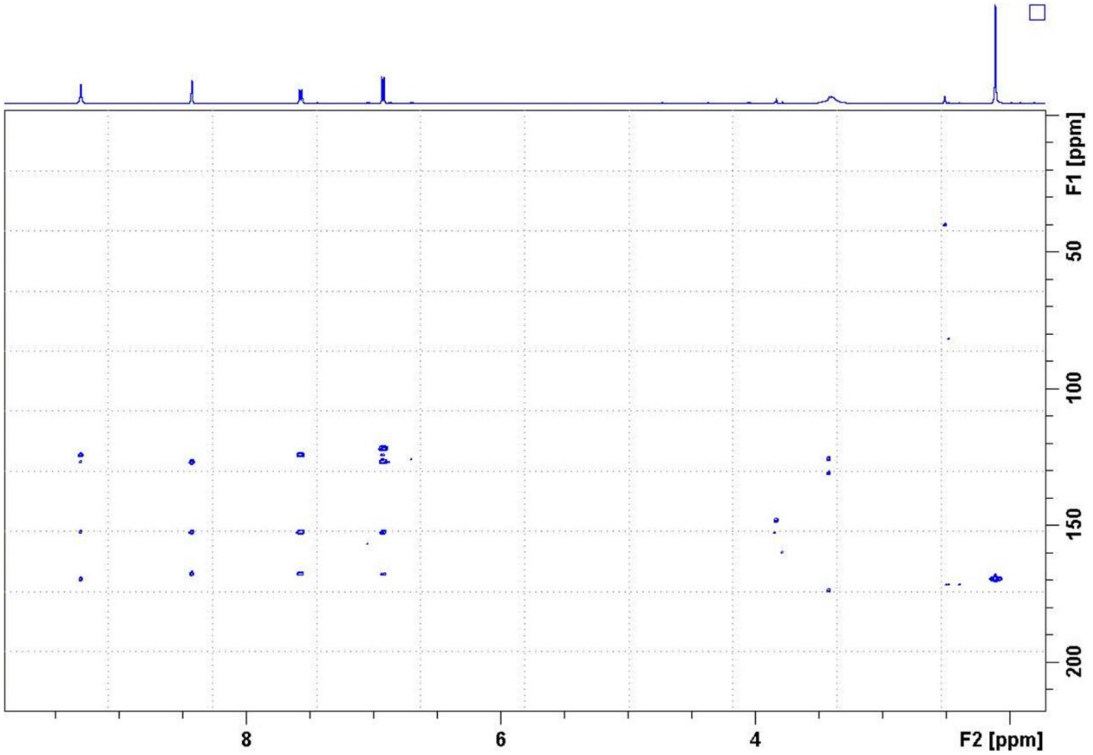

Figure S48. HMBC spectrum of **AHB119**.

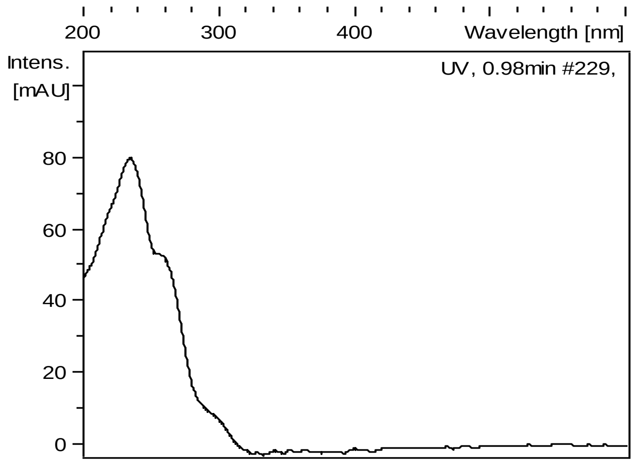

Figure S49. UV-vis (DAD) spectrum of **AHB120**.

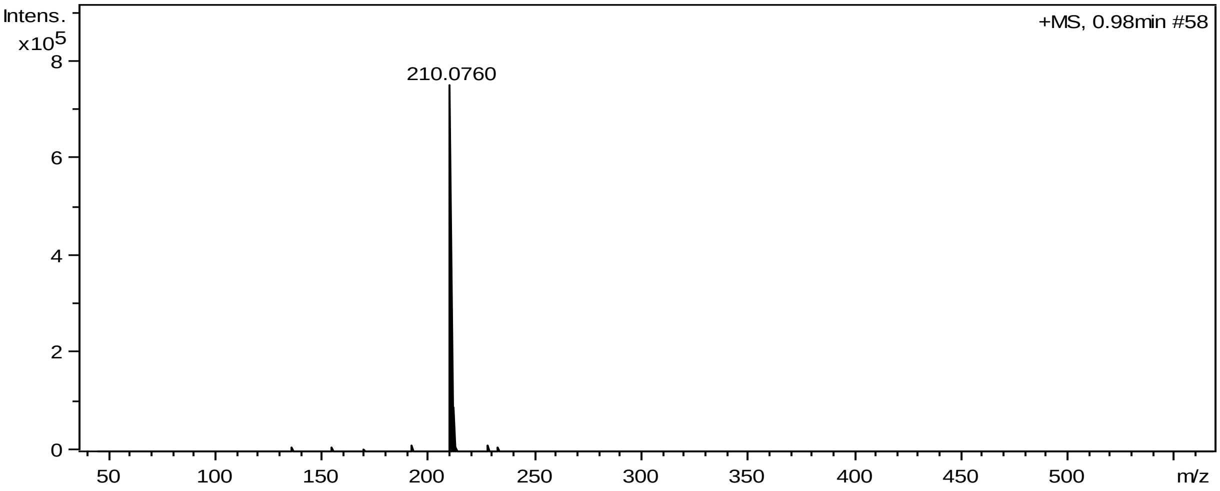

Figure S50. ESI-TOF HRMS spectrum of **AHB120**.

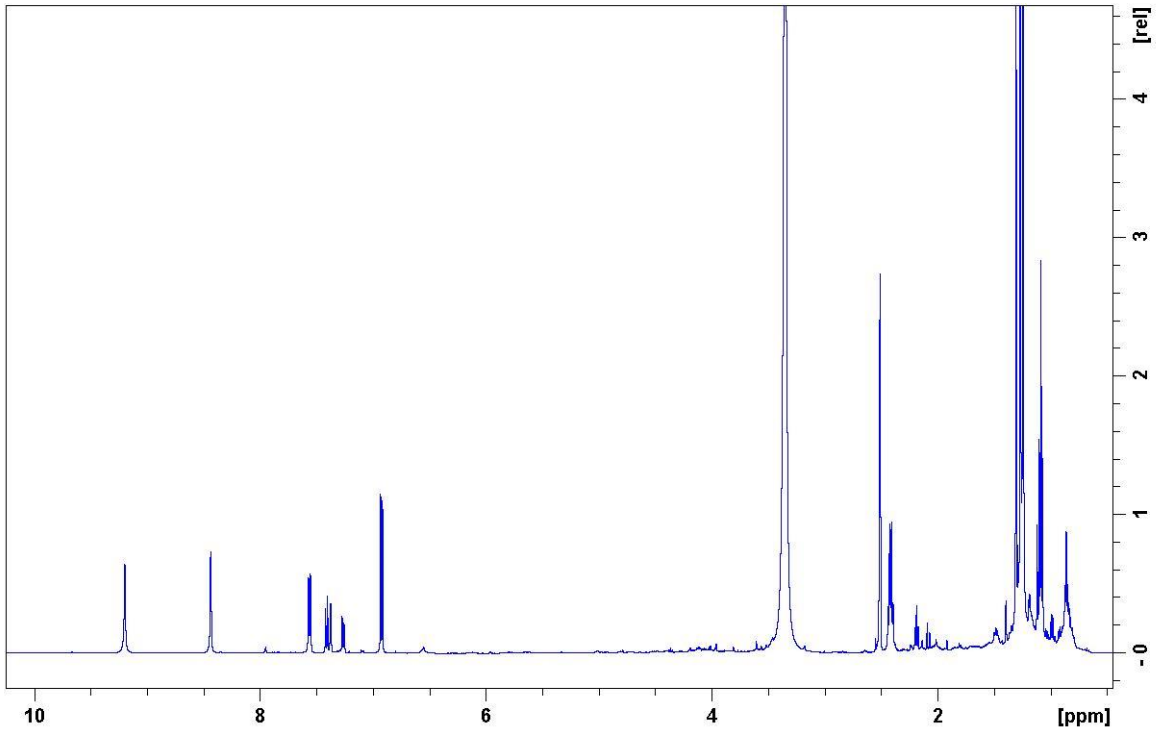

Figure S51. ^{1}H spectrum of **AHB120** (DMSO-d_6, 24 °C, 500 MHz).

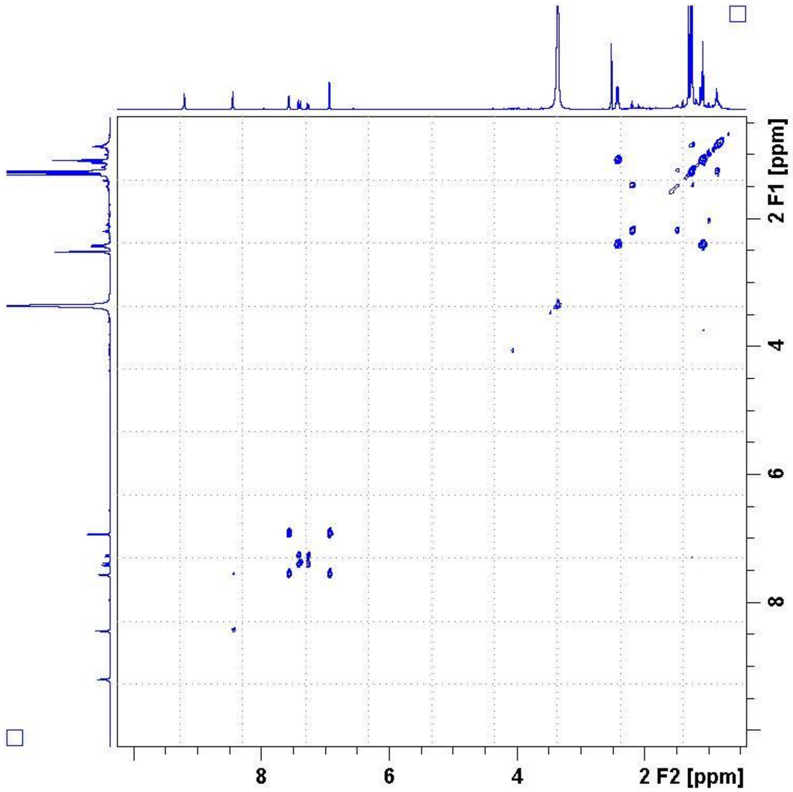

Figure S52. COSY spectrum of **AHB120**.

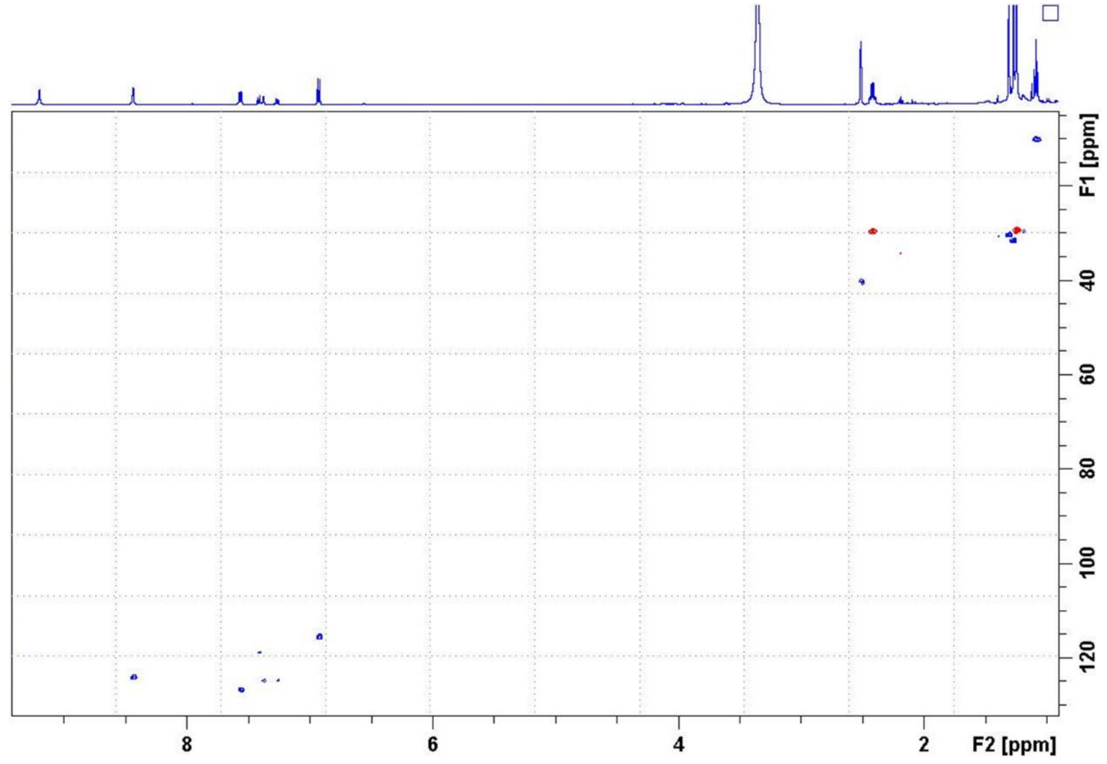

Figure S53. HSQC spectrum of **AHB120**.

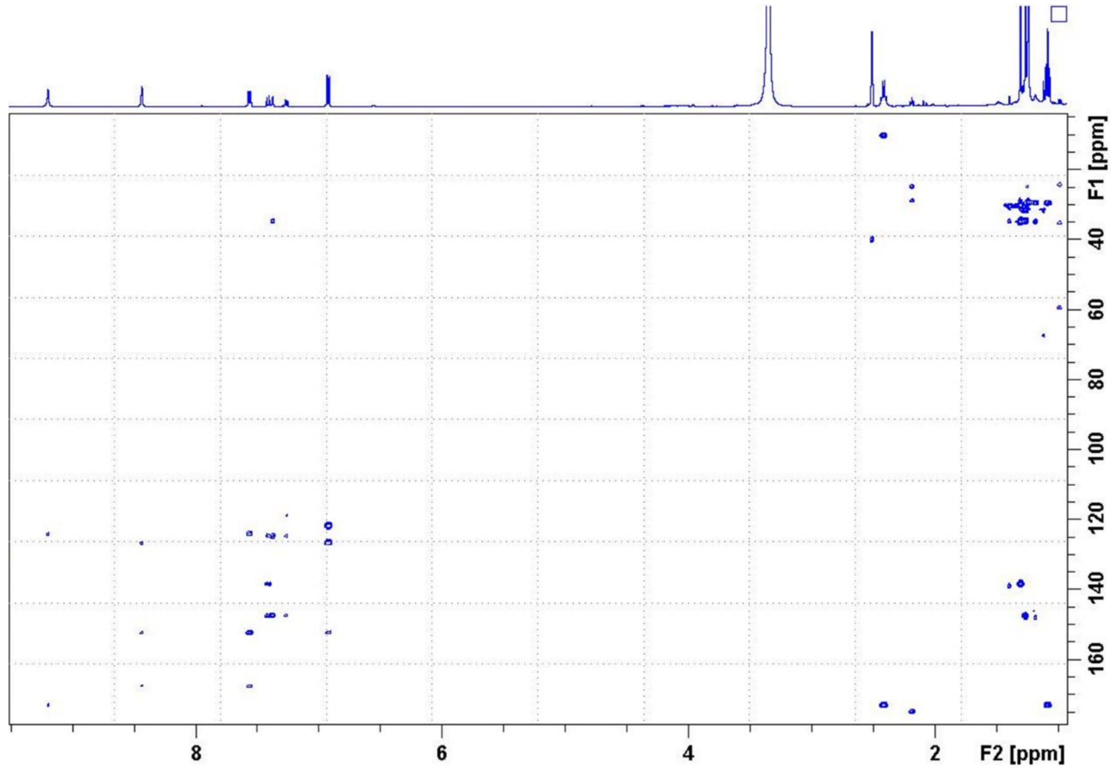

Figure S54. HMBC spectrum of **AHB120**.
